# Genomic and Transcriptomic Analysis of Growth-Supporting Dehalogenation of Chlorinated Methanes in *Methylobacterium*

**DOI:** 10.3389/fmicb.2017.01600

**Published:** 2017-09-01

**Authors:** Pauline Chaignaud, Bruno Maucourt, Marion Weiman, Adriana Alberti, Steffen Kolb, Stéphane Cruveiller, Stéphane Vuilleumier, Françoise Bringel

**Affiliations:** ^1^Department of Molecular Genetics, Genomics, and Microbiology, UMR 7156 Université de Strasbourg (UNISTRA)-Centre National de la Recherche Scientifique Strasbourg, France; ^2^Department of Ecological Microbiology, University of Bayreuth Bayreuth, Germany; ^3^UMR 8030 Centre National de la Recherche Scientifique-CEA, DSV/IG/Genoscope, LABGeM Evry, France; ^4^Institute of Landscape Biogeochemistry-Leibniz Centre for Agricultural Landscape Research (ZALF) Müncheberg, Germany

**Keywords:** dehalogenation, chloromethane, dichloromethane, GEI, genomic island, genome adaptation, methylotrophy

## Abstract

Bacterial adaptation to growth with toxic halogenated chemicals was explored in the context of methylotrophic metabolism of *Methylobacterium extorquens*, by comparing strains CM4 and DM4, which show robust growth with chloromethane and dichloromethane, respectively. Dehalogenation of chlorinated methanes initiates growth-supporting degradation, with intracellular release of protons and chloride ions in both cases. The core, variable and strain-specific genomes of strains CM4 and DM4 were defined by comparison with genomes of non-dechlorinating strains. In terms of gene content, adaptation toward dehalogenation appears limited, strains CM4 and DM4 sharing between 75 and 85% of their genome with other strains of *M. extorquens*. Transcript abundance in cultures of strain CM4 grown with chloromethane and of strain DM4 grown with dichloromethane was compared to growth with methanol as a reference C_1_ growth substrate. Previously identified strain-specific dehalogenase-encoding genes were the most transcribed with chlorinated methanes, alongside other genes encoded by genomic islands (GEIs) and plasmids involved in growth with chlorinated compounds as carbon and energy source. None of the 163 genes shared by strains CM4 and DM4 but not by other strains of *M. extorquens* showed higher transcript abundance in cells grown with chlorinated methanes. Among the several thousand genes of the *M. extorquens* core genome, 12 genes were only differentially abundant in either strain CM4 or strain DM4. Of these, 2 genes of known function were detected, for the membrane-bound proton translocating pyrophosphatase HppA and the housekeeping molecular chaperone protein DegP. This indicates that the adaptive response common to chloromethane and dichloromethane is limited at the transcriptional level, and involves aspects of the general stress response as well as of a dehalogenation-specific response to intracellular hydrochloric acid production. Core genes only differentially abundant in either strain CM4 or strain DM4 total 13 and 58 CDS, respectively. Taken together, the obtained results suggest different transcriptional responses of chloromethane- and dichloromethane-degrading *M. extorquens* strains to dehalogenative metabolism, and substrate- and pathway-specific modes of growth optimization with chlorinated methanes.

## Introduction

Chlorinated one-carbon compounds chloromethane (CM, CH_3_Cl) and dichloromethane (DCM, CH_2_Cl_2_) are toxic chemicals that occur in both pristine and polluted environments. CM is the most abundant halogenated trace gas in the atmosphere, where it contributes to ozone destruction (Carpenter et al., 2014). It is mainly produced by vegetation (Derendorp et al., 2012; Hardacre and Heal, 2013; Rhew et al., 2014; Bringel and Couée, 2015). DCM is one of the most commonly manufactured chlorinated chemicals. It is used for its solvent properties, and is a frequently found contaminant at polluted sites. Some methylotrophic microorganisms are able to utilize chlorinated methanes (such as CM and DCM) as their sole carbon and energy source for growth (Muller et al., 2011a; Nadalig et al., 2014). Bacterial CM and DCM utilization starts with dehalogenation, causing diverse physiological stresses such as production of intracellular HCl, which lowers pH and increases ionic strength, and formation of DNA adducts (Kayser and Vuilleumier, 2001; Torgonskaya et al., 2011; Michener et al., 2014b, 2016). The mechanisms allowing methylotrophic bacteria to cope with dehalogenation-induced stress are still poorly understood, despite potential use of such bacteria for bioremediation.

The aerobic alphaproteobacterium *Methylobacterium extorquens* is the most extensively studied methylotroph. It is found in a wide variety of habitats, including plants, soil, wastewater, and clouds (Amato et al., 2007; Kolb, 2009; Bringel and Couée, 2015). The well-studied *M. extorquens* strains CM4 and DM4, whose genomes have been sequenced (Vuilleumier et al., 2009; Marx et al., 2012), utilize the chlorinated compounds CM and DCM, respectively, as their sole source of carbon and energy. Degradation pathways for CM and DCM have been characterized in these strains, and are also found in many other species (Muller et al., 2011a; Nadalig et al., 2014). The ability to grow on chlorinated methanes has been demonstrated by genetic, biochemical and recent experimental evolution studies to require the expression of essential dehalogenation-associated genes that differ for CM and DCM. To our knowledge no strains able to metabolize both CM and DCM has been isolated so far. Genes *cmuA* and *cmuB* are essential for CM dehalogenation by the *cmu* (CM-utilization) pathway (Vannelli et al., 1999). The two-domain methyltransferase/corrinoid-binding CmuA protein catalyzes methyl transfer from CM to a cobalt-corrin cofactor (Studer et al., 2001), and the methylcobalamin:tetrahydrofolate methyltransferase CmuB transfers the resulting corrinoid-bound methyl group to tetrahydrofolate (H_4_F) (Studer et al., 1999). For each molecule of CM, one methyl-H_4_F and one HCl are produced. For DCM degradation, a glutathione-dependent dehalogenase of the glutathione *S*-transferase family encoded by *dcmA* converts DCM into formaldehyde and two molecules of HCl (Vuilleumier and Leisinger, 1996; Kayser et al., 2002).

Although protons and chloride ions are produced in both cases, the processing of carbon from chlorinated methanes for production of biomass and energy proceeds differently in CM- and DCM-utilizing *M. extorquens* strains (Michener et al., 2016). Thus, toxic chlorinated methanes CM and DCM may generate both similar and compound- and pathway-specific adaptive responses. In this work, we analyzed these responses in terms of global gene expression, by sequencing cDNA libraries of *M. extorquens* strains grown either with CM or with DCM.

## Materials and methods

### Bacterial cultivation and RNA purification

Strains CM4 and DM4 were grown aerobically in 1.2L Erlenmeyer flasks closed with gas-tight screw caps with Mininert® valves (Supelco) in *Methylobacterium* mineral medium (M3) (modified as described in Roselli et al., 2013), with shaking (120 rpm) at 30°C. For 220-mL cultures, one-carbon growth substrates were supplied at 10 mM final concentration, by adding either aqueous solutions of 2.75 mL of filter-sterilized aqueous stock solution of methanol (800 mM), 141 μL of neat dichloromethane, or 40 mL of gaseous chloromethane (assuming a Henry constant of 0.0106 m^3^⋅atm⋅mol^−1^ at 30°C) (Chen et al., 2012). Upon reaching mid-exponential phase (OD_600_ ~0.15), growth was stopped by addition of 27.5 mL stabilization buffer. This buffer was prepared by mixing 5 mL of phenol and 5 mL of 1 M sodium acetate pH 5.5, then after centrifugation at 1,800 g for 3 min, 5 mL of the lower phase was mixed with 95 mL of absolute ethanol. Resulting cell suspensions were centrifuged at 5,000 rpm for 5 min at 4°C, and suspended in 10 mL TE containing 2 mg⋅mL^−1^ lysozyme (Euromedex). After 15 min incubation at 37°C, each cell suspension was centrifuged 10 min at 4°C, the obtained pellet resuspended in 10 mL of Trizol (Invitrogen), and 2.5 mL of chloroform was added. RNA was precipitated with isopropanol and washed with ethanol (70%), then resuspended in DEPC-water and treated with DNAse (Turbo DNAse, Invitrogen). DNA depletion was checked by PCR (see Table S1 for primers). RNA quality was checked with the RNA 6000 Nano kit on a Bioanalyzer 2100 (Agilent Technologies), and quantified with the Qubit RNA kit (Invitrogen). Depletion of rRNA was obtained by treating 5 μg of total RNA with the Gram-negative RiboZero Magnetic kit (Tebu-Bio) according to the manufacturer's protocol.

### cDNA library preparation, sequencing and data normalization

rRNA-depleted RNA (50–60 ng in 5 μL) were fragmented by adding 13 μL of the “Fragment, prime, finish mix” of the “Purify and fragment mRNA” kit (Illumina). Then, cDNA libraries were constructed with the TruSeq stranded mRNA LT kit (Illumina) following the manufacturer's protocol. Obtained cDNA libraries were quality checked (Bioanalyzer, DNA 1000 kit, Agilent Technologies) prior to HiSeq2000 sequencing. Paired-end sequence mapping was used to limit mapping artifacts and remove mapping ambiguities between gene paralogs. Factor size normalization of the raw counts was performed using a set of 55 reference “housekeeping” genes instead of the standard method using the complete set of CDS (Anders et al., 2013), as a few genes had very high read numbers in growth with chlorinated methanes only. The 55 reference genes cover a wide range of expression levels but had unchanged transcript abundance in the tested biological replicates (log_2_fc value between −0.9 and 1.2) (Table S2). Normalization was validated by comparison with the complete set of CDS for the methanol dataset (Figure S1). Gene transcripts were defined as differentially abundant when the log_2_ of fold-change values (log_2_fc) between cultures grown with chlorinated methanes and methanol were ≥2.0 or ≤ −2 (Yang et al., 2015). Each condition was analyzed in duplicate, with the average of read sense and antisense for each condition. Data were considered significant when False Discovery Rate (i.e., FDR) was ≤0.1 as previously described (Benjamini and Hochberg, 1995). The complete RNA-Seq dataset is accessible online (https://www.genoscope.cns.fr/agc/microscope/transcriptomic/NGSProjectRNAseq.php?projType=RNAseq).

### RT-qPCR

Retrotranscription was performed from 2.0 μg of DNA-depleted RNA extracted using the Nucleospin RNA plus kit (Macherey Nagel) combined with Turbo DNase and Turbo DNA free kit (Invitrogen) using Superscript III (Invitrogen) and random hexanucleotide primers (Invitrogen) following manufacturers' protocols, except that RNaseOUT™ was replaced by RNasin™ (40 U⋅μL^−1^; Invitrogen), with inclusion of appropriate controls (reactions without RNA template or Superscript III enzyme). Amplification was performed in qPCR 96 wells plates (Agilent Technologies) covered with Microseal B adhesive seals (Bio-Rad) using the Thermocycler Stratagene Mx3005P (MxPro software v4.10; Agilent Technologies). Master mix containing 7.5 μL Brilliant III SYBR™ Green low ROX qPCR master mix (Agilent Technologies), 0.225 μL of each primer (Eurofins) at 20 μM and 3.3 μL molecular biology grade water was mixed with 3.75 μL template cDNA. After 3 min pre-denaturation at 95°C, 40 cycles of 20 s at 95°C and 20 s at 60°C, a final one-cycle step of 1 min at 95°C with ramping from 60 to 95°C was applied to obtain dissociation curves for quality control of PCR products. For each biological triplicate, technical duplicates were analyzed and compared to standard curves with gDNA (0.1–1⋅10^−5^ ng⋅μL^−1^) and a no template control. SYBR™ green fluorescence data normalized with Rox fluorescence were analyzed using the package qpcR (v1.40) within R for Ct determination (Ritz and Spiess, 2008). Fold change values were calculated by the 2^−ΔΔCt^ method (Schmittgen and Livak, 2008), with *rrsA* as internal control and *dcmA* in DM4 cultures grown with methanol as the reference condition.

### Comparative genomics analysis

Comparative analysis of *M. extorquens* genomes was done in MaGe on the Genoscope MicroScope online platform (Vallenet et al., 2017), with the sequenced genomes of 5 strains of *M. extorquens*, i.e., the 2 dehalogenating strains CM4 (Genbank accession numbers CP001298, CP001299, CP001300) and DM4 (FP103042, FP103043, and FP103044), and 3 other strains AM1 (CP001511, CP001512, CP001513, and CP001514), PA1 (CP000908), and BJ001 (CP001029, CP001030, and CP001031) (Vuilleumier et al., 2009; Marx et al., 2012). All studied strains contain at least one plasmid, except for PA1. Proteins responsible for dehalogenation of CM or DCM are exclusively encoded by *M. extorquens* CM4 and DM4 genomes, respectively. The common genome called hereafter “core” was operationally identified using the MaGe Pan/Core-genome tool (https://www.genoscope.cns.fr/agc/microscope/compgenomics/pancoreTool.php?), by defining shared CDS as encoding proteins displaying at least 80% amino acid identity over 80% of CDS length in all considered genomes. Other genes were assigned either to the variable genome when present in at least 2 genomes, to the dehalogenation-associated genome when shared only by strains CM4 and DM4, and to the strain-specific genome when found only in one genome, respectively. Genomic islands of at least 5 kb were defined using the MaGe “Regions of Genomic Plasticity” tool (https://www.genoscope.cns.fr/agc/microscope/compgenomics/genomicIsland.php?) with the genomes of the 5 strains above, and applying a specificity score cutoff of 40 for all compared genomes.

## Results

Acquisition of specific dehalogenase genes by *M. extorquens* does not necessarily lead to growth with chlorinated methanes (Kayser et al., 2002; Michener et al., 2014a,b, 2016). Adaptation may require other specific genes associated with dehalogenative pathways, as well as modulation of expression of common “household” genes to optimize metabolic flux and responses to dehalogenation-associated stresses. In this study, the relative contribution of core, variable and strain-specific genomes in *M. extorquens* strains growing with halogenated methanes was investigated using a combination of comparative genomics and transcriptomics.

### The potentially dehalogenation-associated genome of *M. extorquens* is limited

**Figure 1:**
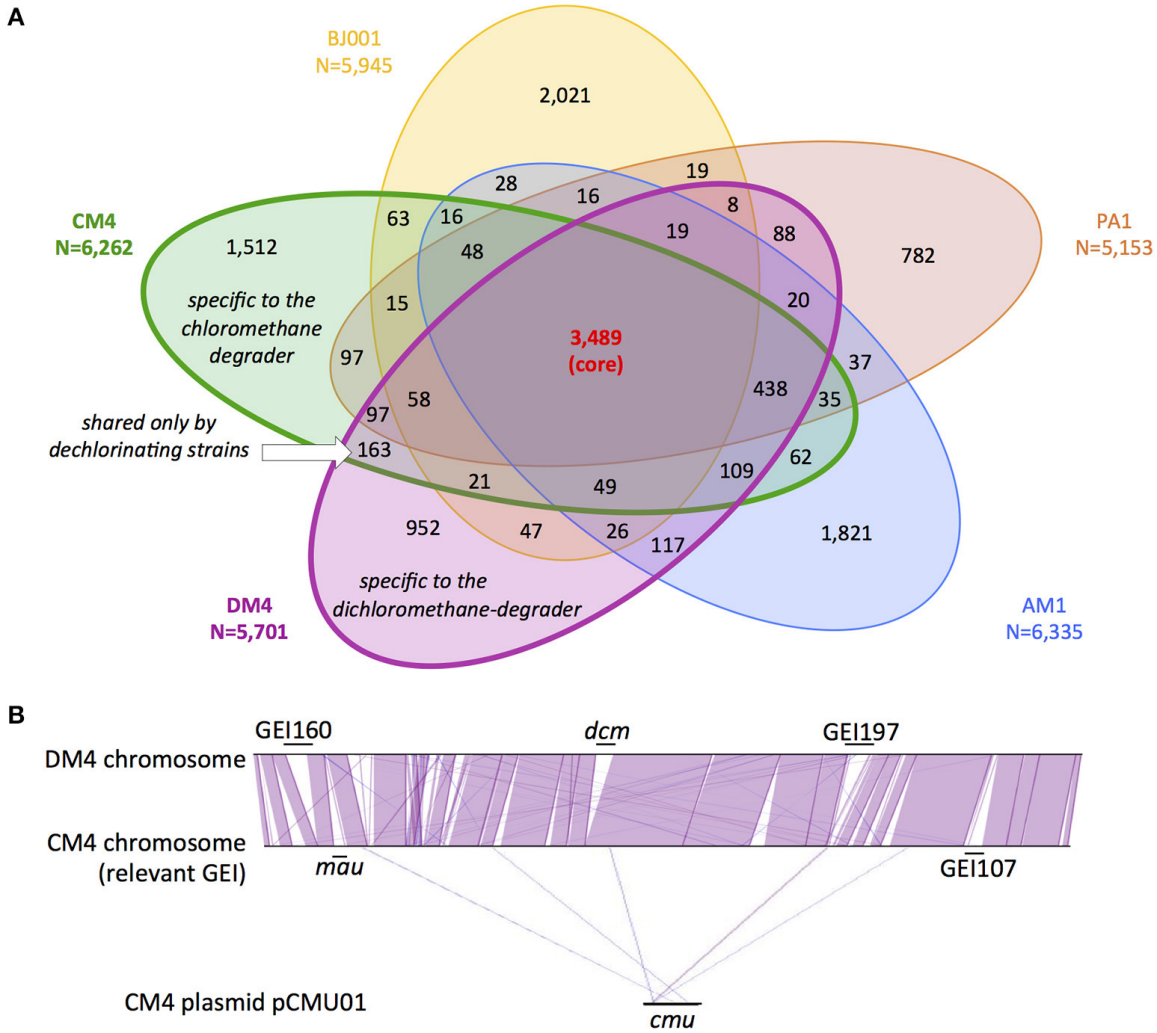
Gene content and synteny in genomes of *M. extorquens* CM4 and DM4. Comparative genome analyses were performed on the sequenced and assembled genomes of *M. extorquens* dehalogenating strains CM4 and DM4, as well as strains AM1, PA1, and BJ001 (Vuilleumier et al., 2009; Marx et al., 2012), using MaGe (Vallenet et al., 2017) on the MicroScope online platform (see Materials and Methods). **(A)** Common homologous genes were defined and assigned to either core (found in all 5 strains), variable (found in 2–4 strains), shared (found only in CM4 and DM4) or specific (found in one strain only) genomes. **(B)** Synteny of CM4 and DM4 genomes indicating genomic islands (GEI) relevant to C_1_ metabolism, i.e., *dcm* for dichloromethane utilization and *mau* for methylamine utilization (Vuilleumier et al., 2009), and *cmu* for CM utilization (380 kb plasmid pCMU01, Marx et al., 2012) (see Table 3 for GEI details). Synteny involved groups of at least 6 homologous genes (purple lines, strand conservation; blue lines, strand inversion).

Beyond specific genes associated with dehalogenation [at least 6 genes of the *cmu* pathway for strain CM4 (Michener et al., 2016), and 4 genes of the *dcm* islet for strain DM4 (Muller et al., 2011a, respectively)], few strain-specific genes shared by CM- and DCM-dehalogenating strains were identified through comparison with 3 other high quality assembled genomes from *M. extorquens* (Figure 1A). The strains AM1, PA1, and BJ001 were experimentally checked for their inability to grow on chloromethane or dichloromethane (data not shown). The gene content in the 5 *M. extorquens* genomes totals 12,273 unique CDS, representing 12.3 Mb. The core genome shared by all 5 *M. extorquens* genomes is extensive (3,489 CDS) ranging from 55 to 68% (55% for CM4; 61% for DM4). *M. extorquens* strains CM4 and DM4 share most of their genome content (75%, 4,424 CDS) and extensive gene synteny (Figure 1B). The specific genome for strain CM4 comprises 1,512 CDS (24% of total), and that of strain DM4 952 CDS (17%), respectively. In contrast, the variable genome shared only by the 2 dehalogenating strains was only 163 CDS, representing less than 3% of their total genome size, and in the range of the shared variable genome for any pair of the 5 strains considered (Figure 1A). A majority (97 CDS) had no predicted function and 42 genes were found in synteny on plasmids pCMU01 in CM4 and plasmid p1METDI in DM4 (Table S3).

### Common transcriptomic response of *M. extorquens* strains to chlorinated methanes as growth substrates

*Methylobacterium extorquens* strains CM4 and DM4 were grown with CM and with DCM, respectively. Longer generation times were observed for growth with CM and DCM compared to with methanol, as observed previously (Muller et al., 2011b; Roselli et al., 2013). Global profiles of gene expression were assessed by RNA-Seq with sequencing coverage exceeding 600X (Table 1). Only 29 CDS of strain CM4 and 36 CDS of strain DM4 showed no reads. Obtained data were normalized (Table S1; Figure S1) and verified by qPCR for a set of 16 genes spanning 2 orders of magnitude of gene expression (Table S2; Figure S2). Only genes with satisfactory false discovery rates (*p*-value < 0.1) were further analyzed and included 1,245 and 301 genes for the genomes of strain CM4 and strain DM4, respectively.

**Table 1:** Overview of genomic properties of *M. extorquens* CM4 and DM4 and obtained RNA-Seq data.

**Strain**	**Genome^a^**	**Growth condition generation time (h)^b^**	**Total reads^c^**	**Mapped reads (%)**	**rRNA (%)^d^**
CM4	chromosome 5.8 Mb GC% = 68.2 pCMU01 380.2 kb GC% = 66.3 p2MCHL 22.6 kb GC% = 63.9	Methanol (3.0 ± 0.2)	30,337,270 37,414,003	98.6 98.2	18.3 14.3
		Chloromethane (5.4 ± 0.4)	53,790,411 36,665,352	96.3 97.5	18.6 25.4
DM4	chromosome 5.9 Mb GC% = 68.1 p1METDI 141.5 kb GC% = 65.3 p2METDI 38.6 kb GC% = 63.7	Methanol (3.4 ± 0.4)	48,154,448 38,757,418	99.1 97.9	12.3 23.0
		Dichloromethane (9.0 ± 0.7)	43,101,981 32,066,920	95.0 95.0	34.7 20.0

a *In strain CM4, the CM utilization pathway is encoded by cmu genes located on plasmid pCMU01 (Roselli et al., 2013). In strain DM4, dcm genes involved in DCM utilization are located on 5.5 kb dcm islet (Muller et al., 2011b) within a 126 kb genomic island on the chromosome (Vuilleumier et al., 2009)*.

b *Aerobic growth in M3 medium with 10 mM one-carbon substrate provided as sole source of carbon and energy*.

c *Illumina HiSeq2000*.

d *Percentage of total reads*.

**Figure 2:**
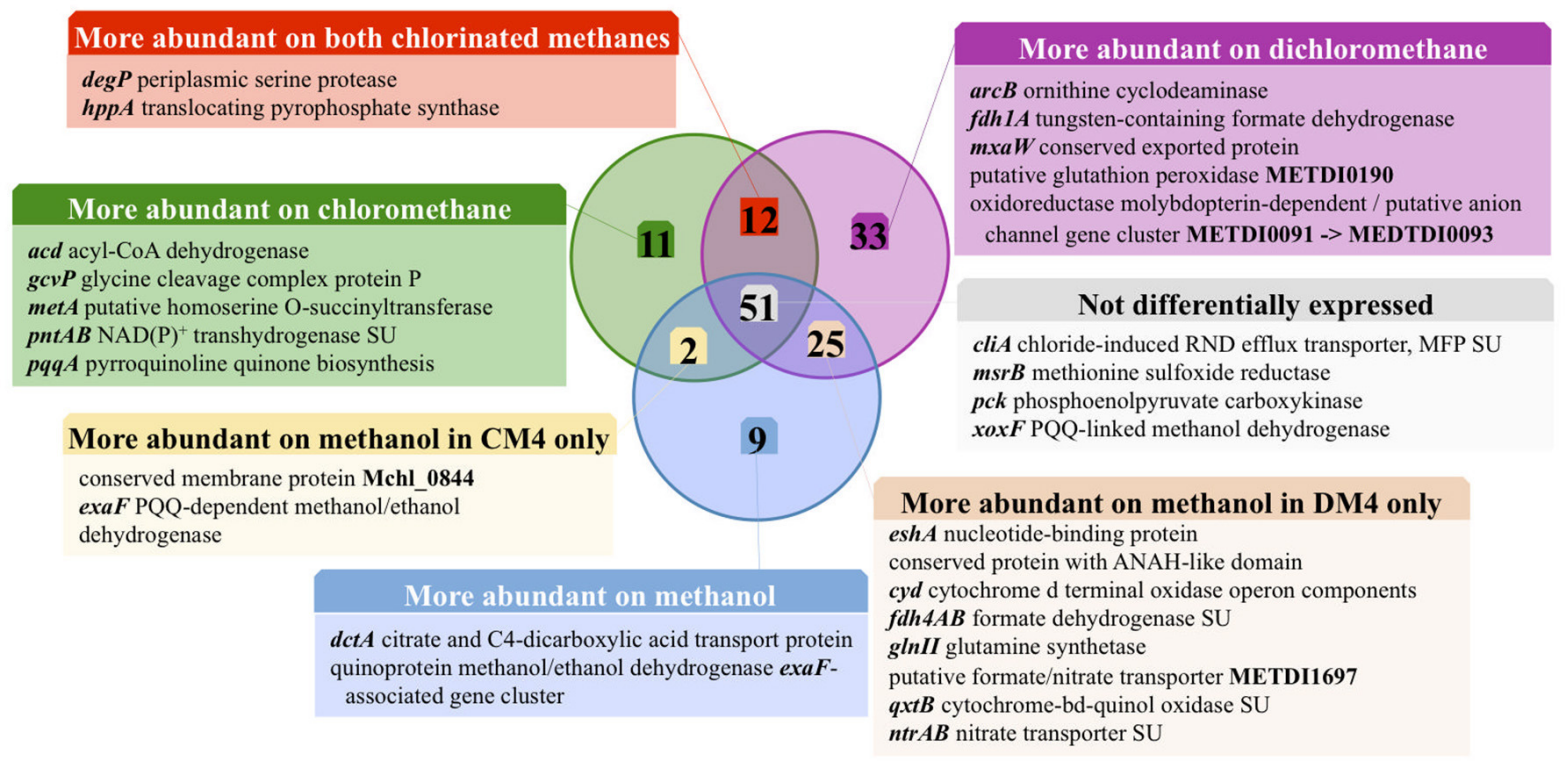
Carbon source-dependent transcript abundance of common core genes in *M. extorquens* CM4 and DM4. Different categories were defined on the basis of the log_2_ fold-change (log_2_fc) values of RNA-Seq reads for *M. extorquens* CM4 grown with CM (green circle) and for *M. extorquens* DM4 grown with DCM (purple circle) versus methanol (blue cercle). Complete gene names, log_2_fc values for each category are detailed in Table S4.

Transcript abundance of strains grown with chlorinated methanes CM or DCM and with methanol, the reference methylotrophic growth substrate for *M. extorquens*, were then compared. A total number of 150 genes for CM4 and 190 genes for DM4 were detected as differentially abundant genes (Table 2). Overall gene expression of common genes during growth with methanol was similar in strains CM4 and DM4, as expected (Figure S1). More pronounced differences were observed between patterns of gene expression for common genes of *M. extorquens* strains CM4 and DM4 grown with CM or with DCM (Table S4), with only few genes showing the same trend with CM and DCM relative to methanol (Figure 2). Strikingly, essentially all 163 CDS only shared by the 2 dehalogenating strains and not found in any of the 3 other non-dehalogenating *M. extorquens* strains (Figure 1A) lacked differential expression between chlorinated methanes and methanol (with the exception of METDI4814, less abundant with DCM) (Table S3; Figure S3B). Among those, 42 genes were plasmid-borne and co-localized on the largest plasmids in both strains (Table S3), with some displaying high transcript abundance (Figure S3).

**Table 2:** Differential expression of core, variable, shared and strain-specific CDS during growth with chlorinated methanes.

**Strain**	**Genome^a^**	**CDS number**	**Differential transcript abundance with chlorinated methanes**		
			**Ratio (%)**	**Higher^b^**	**Lower^b^**
CM-degrading *M. extorquens* CM4	Core	3,489	1.6	45	11
	Variable	1,098	1.8	19	1
	Shared only with DM4	163	0	0	0
	Specific to CM4	1,512	4.9	73	1
DCM-degrading *M. extorquens* DM4	Core	3,489	1.8	31	32
	Variable	1,097	5.9	28	46
	Shared only with CM4	163	0	0	0
	Specific to DM4	952	5.6	10	43

a *Common, variable, shared only by dehalogenating strains CM4 and DM4, and strain-specific genomes, as defined in Material and Methods*.

b *Number of CDS with higher (log_2_fc > 2) or lower (log_2_fc < −2) transcript abundance in cultures grown with chlorinated methanes compared to with methanol*.

Of the 12 common genes encoded by the core genome more highly expressed during growth with either CM and DCM than with methanol (Table S4; Figure 2), only 2, *degP* and *hppA*, have predicted functions. Gene *degP* encodes a putative periplasmic serine protease whose *Escherichia coli* homolog HtrA (39% amino acid identity) is a central housekeeping molecular chaperone protein controlling the production of functional, properly folded outer-membrane proteins (Ge et al., 2014). In *Carboxydothermus hydrogenoformans*, the membrane-bound proton translocating pyrophosphatase HppA (48% amino acid identity to *M. extorquens* homologs) uses energy from pyrophosphate hydrolysis to build up a proton motive force by proton extrusion (Belogurov and Lahti, 2002). In *Rhodospirullum rubrum*, the closely homologous HppA (72% amino acid identity) is involved in stress bioenergetics and in particular salt stress (Lopez-Marques et al., 2004).

The number of genes of the core genome with significantly lower expression on chlorinated methanes is also low (9 genes, Figure 2). These may rather be methanol-induced rather than genes repressed by chlorinated methanes, since 7 of these genes belong to predicted operons associated with the alternative alcohol dehydrogenase ExaF to the paradigmatic methanol dehydrogenase encoded by *mxa* genes (Good et al., 2016; Table S4).

**Figure 3:**
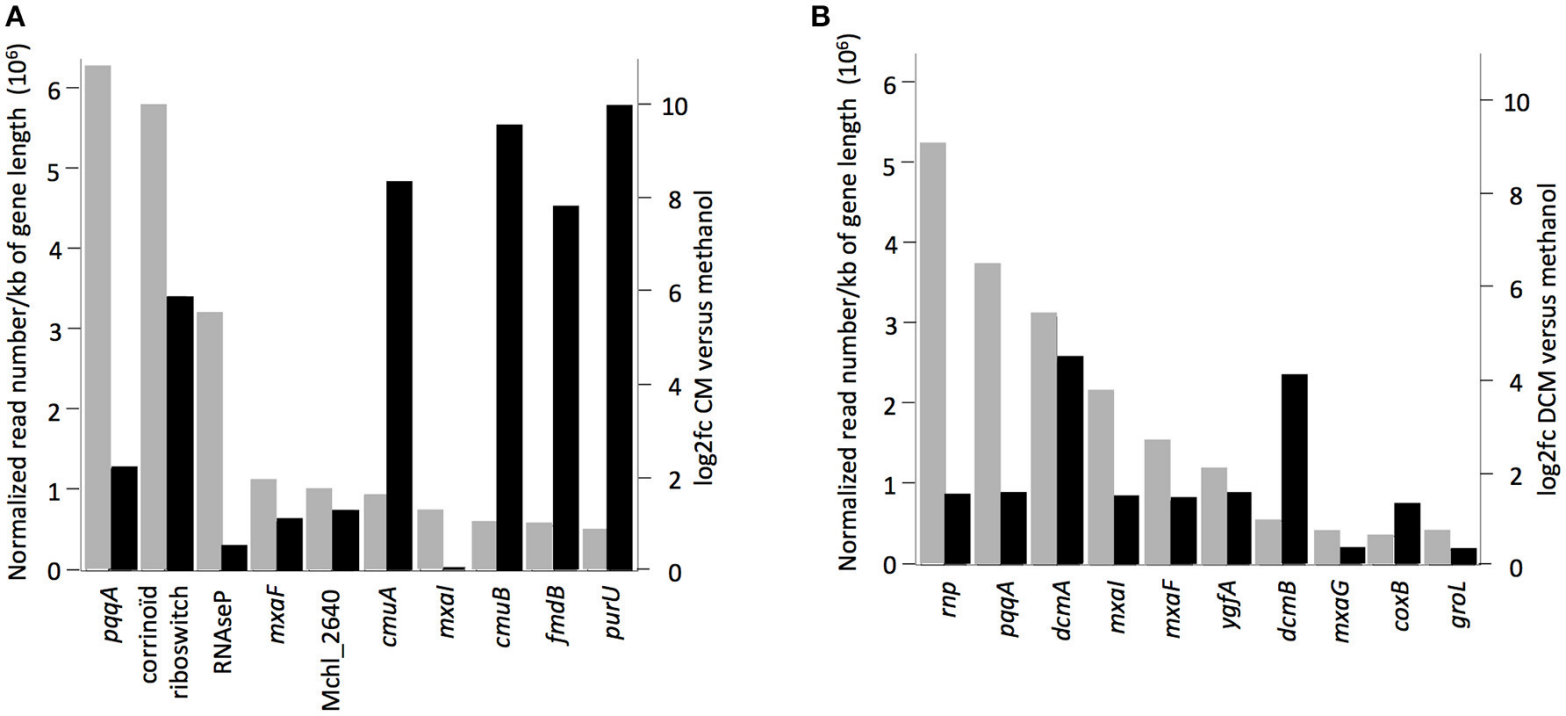
Genes with highest transcript abundance in cultures grown with chlorinated methanes compared to with methanol. Black rectangles indicate values of log_2_fc (values higher than 2.0 or lower than −2.0 mean normalized reads more abundant in cultures grown with chlorinated methanes or with methanol, respectively). Gray rectangles represent normalized read numbers divided by gene length in kb. RNA-Seq data from cultures of **(A)** *M. extorquens* CM4, **(B)** *M. extorquens* DM4.

The 10 most highly transcribed genes during growth with chlorinated methanes in strains CM4 and DM4 (Figure 3) included 3 common to both strains, albeit with values of log_2_fc <2 compared to growth with methanol. Of the other 7 genes highly transcribed on chlorinated methanes, only strain-specific genes directly associated with dehalogenation (5 for strain CM4, 2 for strain DM4), and not common genes, showed log_2_fc values >2. Transcription responses for growth with CM and DCM were analyzed in more detail.

### The chloromethane transcriptome of strain CM4

**Figure 4:**
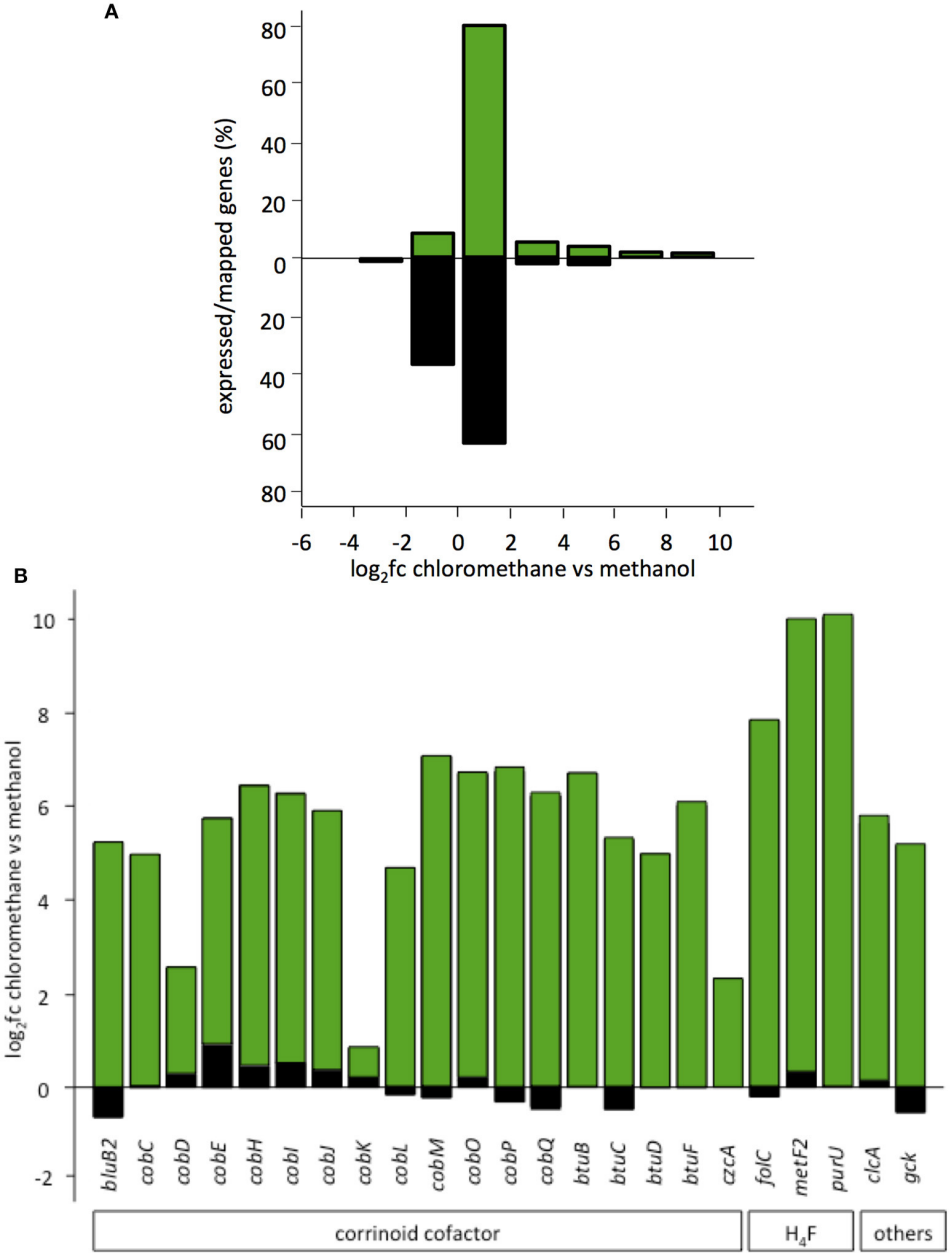
Involvement of plasmid pCMU01 in the chloromethane transcriptome. **(A)** Expressed percentage of total genes per log_2_fc range. Genes encoded by plasmid pCMU01 (in green) and by the *M. extorquens* CM4 chromosome (in black) were grouped according to their log_2_fc values. Chromosome and plasmid pCMU01 encode 6,262 and 361 genes, and the total percentage of expressed genes is 1.4 and 17.5%, respectively. **(B)** Differential expression of pCMU01- and chromosome-encoded paralogs.

Under the conditions tested, a total of 137 genes (2% of the CM4 transcriptome) had higher transcript abundance with CM than with methanol. Only 43 of these belong to the core genome defined here for *M. extorquens* (log_2_fc ≥ 2; Table S5; Figure 4A). Among these, 11 core genes only differentially expressed in strain CM4 had not previously been associated with *M. extorquens* growth with CM. They include the *pnt* gene cluster encoding a NADH/NADPH transhydrogenase with cross-membrane proton translocation activity (Chou et al., 2015); two consecutive paralogs of *pqqA*, a precursor of the redox active dehydrogenase cofactor PQQ (Ochsner et al., 2015); *ykuD*, encoding a transpeptidase of a large enzyme family associated with cell wall biology (Bielnicki et al., 2005); *ada* encoding a bifunctional transcriptional activator which acts in response to alkylation damage (51% amino acid identity with the well-characterized *E. coli* homolog) (McCarthy and Lindahl, 1985); *ibpA* encoding a small heat shock protein (sHSP) with 57% amino acid identity to *E. coli* IbpA, which protects enzymes against oxidative stress (Kitagawa et al., 2002).

**Figure 5:**
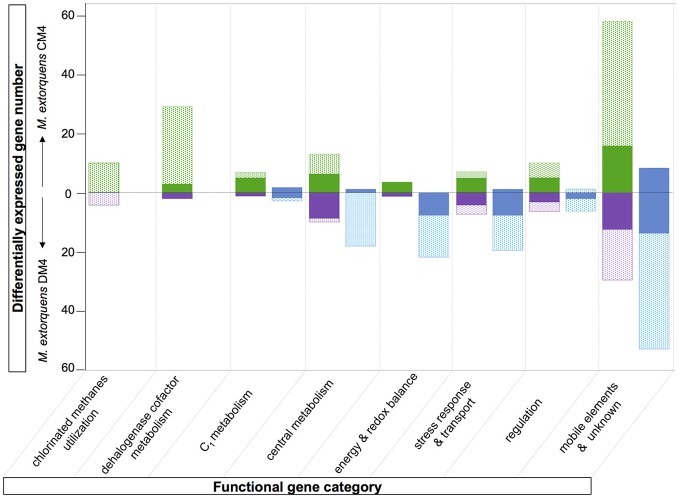
Overview comparison of the chloromethane and dichloromethane transcriptomes. Differentially abundant genes with predicted function are clustered in different functional categories (see Tables S6, S7). The fractions of genes of the core and variable genomes are indicated in plain and dotted rectangles, respectively. Transcript with higher abundance in chloromethane, dichloromethane or methanol are in green, purple or blue, respectively.

The major contribution in the CM transcriptome involves strain-specific genes either directly or indirectly associated with dehalogenation (Figure 5). First, 40 genes known from previous work to be associated with *cmu* gene clusters in CM-degrading strains, and found on the pCMU01 plasmid in strain CM4, showed highest relative expression (log_2_fc values 7.7–10) during growth with CM (Table S5; also see Figure 4A). Most of these genes are involved in metabolism and transport of the corrinoid and H_4_F cofactors essential for CM dehalogenation by the *cmu* pathway (Studer et al., 2001) (Table S5; Figure 4B). A gene cluster specifically shared between sequenced genomes of CM degrading isolates [acxABC, Roselli et al., 2013 >81% amino acid identity with the characterized aerobic bacterium *Xanthobacter autotrophicus* acetone carboxylase components (Sluis et al., 2002)] was also more expressed in cells grown with CM (see Figure S3A for log_2_fc values and expression level) although its functional connection with the *cmu* pathway remains to be characterized. Second, half of the plasmid pCMU01-borne genes more highly expressed on CM had homologous copies located on the chromosome (31 genes out of 62 plasmid-borne genes with higher transcript abundance; Table S5; Figure 3B). All the corresponding chromosomal paralogs were, in contrast, not differentially expressed. Again, a majority of plasmid-borne homologs were associated with the essential corrinoid and tetrahydrofolate cofactors of CM dehalogenation by the *cmu* pathway (Figure 3B). Similarly, the pCMU01 plasmid homolog of the *clc* H^+^/Cl^−^ antiporter gene was more highly expressed on CM (log_2_fc value of 5.5), unlike its chromosomal homolog (68% identity at the protein level; Figure S3A). Taken together, this argues strongly for a key role of plasmid pCMU01-borne genes in adaptation to growth with CM in strain CM4.

### The dichloromethane transcriptome of strain DM4

Under the conditions tested, only 3% of the DM4 transcriptome was altered in growth with DCM relative to methanol. Among these, 69 genes (1% of the transcriptome) showed higher transcript abundance in cultures grown with DCM (Table 2). These included the 4 genes of the *dcm* islet, i.e., *dcmA* coding for the DCM dehalogenase essential for growth with DCM; *dcmR* involved in its own transcription as well as that of *dcmA* (Leisinger et al., 1994; Kayser et al., 2002); and *dcmB* and *dcmC* genes of unknown function (Table S6). This is the first reported evidence for transcription of *dcmC* and its modulation by DCM, and confirms previous results for *dcmB* (Muller et al., 2011b). Transcripts of *dcmA* and *dcmB* were among the most abundant with DCM (Figure 3B).

The majority of genes showing differential abundance with DCM only (33 genes) belong to the core genome of *M. extorquens*, and significantly exceeds the number of *M. extorquens* genes of the core genome also more highly abundant during growth with CM (12 genes), or those only more highly expressed during growth with CM but not DCM (11 genes) (Figure 2). The specific functions of most of these genes remain elusive, although an association with redox status is suggested, with a putative glutathione peroxidase (METDI0190) and 2 sets of genes associated with uncharacterized molybdenum-dependent enzymes (METDI2693, METDI0091- METDI0093) showing increased transcript abundance (Table S6). Of those, the gene encoding the glutathione peroxidase was highly expressed (Figure S3B).

The contribution of the 2 plasmids of DCM-degrading strain DM4 (Table 1) in the DCM transcriptome is limited (2 uncharacterized genes among 186 CDS were differentially expressed). This suggests that unlike for the CM-degrading strain, DM4 plasmids do not play a role in adaptation to DCM in strain DM4.

The set of genes from strain DM4 with higher expression on methanol is also larger than that of strain CM4 (Figure 5). The majority of these genes is found in gene clusters with redundant gene content (Figure S4), and often associated with genomic islands (GEIs) (Table 3, and see next subsection).

**Table 3:** GEIs containing genes with differential transcript abundance.

**GEI^a^**	**Length (kb)**	**CDS (start-end)**	**Structure^a^**	**Number**		**% CDS of GEI**	**Genes and potential relevant functions**
				**CM or DCM**	**Methanol**		
**STRAIN DM4**							
*dcm*	126	METDI2550–METDI2682	(none) tRNA-int-mob-misc_RNA-SIGI-AH (none)	4	0	3	DCM utilization (*dcmRABC*)
GEI160	160	METDI0225–METDI0426	(tRNA)-int-SIGI-AH (int)	1	35	18	Carbon metabolism (*ackA*-like, *adh*-like, *xfp, fabI, phbC*); energy (ATPase, cyt. c, cyt. c oxidase); stress (*clpB, DnaJ*)
GEI197	197	METDI4320–METDI4570	(none) tRNA-int-mob-SIGI-AH (IS)	1	34	14	Carbon metabolism (*ackA*-like, *adh*-like, *xfp*); energy (ATPase, cyt. c, cyt. c oxidase); members of ANAH-like superfamily
**STRAIN CM4**							
GEI107	107	Mchl4758–Mchl4844	tRNA-int-SIGI-IVOM-Specific_Region	4	0	5	Putative porin; others without predicted function
pCMU01^b^	194	Mchl5386–Mchl5736	Unknown	63	0	18	CM utilization (*cmu*); corrinoid cofactor biosynthesis (*cob, czc, bluB2*); H_4_F cofactor metabolism (*bluB2, folC2*); carbon assimilation (*acx*)

a *Identified with “Regions of Genomic Plasticity” tool (MaGe plateform, Vallenet et al., 2017 using M. extorquens CM4, DM4, AM1, PA1, and BJ001 genomes). Int, integrase; mob, mobility determinant; AH, region rich in genes detected by Alien Hunter based on variable-length k-mers bias (Vernikos and Parkhill, 2006); SIGI, region with biased codon usage (Waack et al., 2006)*.

b *Plasmid pCMU01 (380 kb, 350 predicted CDS) was only found so far in M. extorquens CM4 (Roselli et al., 2013), precluding analysis with the RGP tool except to remove extensive regions of synteny with DM4 plasmid p1METDI (Mchl5546-Mchl5579, Mchl5591-Mchl5621 in plasmid pCMU01). Hence, the 239 CDS without hits to the other 4 M. extorquens genomes were conservatively defined maximal GEI*.

### Expression of genomic island-borne genes in dehalogenating *M. extorquens* strains

As plasmids, GEIs are major agents of rapid genome evolution and adaptation of catabolic pathways in bacteria, and are often found integrated in the chromosome (van der Meer and Sentchilo, 2003). The potential role of GEIs in adaptation of *M. extorquens* to growth with chlorinated methanes was investigated. Out of the 14 and 11 specific GEIs detected in strains CM4 and DM4, respectively, only 3 GEIs contained genes with differential expression (Table 3). Such genes only represented a minor part of GEIs, and their roles beyond that of genes associated with dehalogenation remain unknown. It is striking that no other genes of the 126 kb *dcm* GEI of strain DM4 were differentially transcribed beyond those of the 5.5 kb *dcm* islet, unlike many genes associated with *cmu* gene clusters on plasmid pCMU01 (Table 3).

## Discussion

### Dehalogenation-specific gene complement

The number of strain-specific genes, as such potentially associated with dehalogenative metabolism, was relatively small (Figure 1A) and genes with an annotation suggestive of a role in this context were not detected (Table S3). Moreover, apart from dehalogenase genes, such genes were not especially prone to differential expression with chlorinated methanes (Tables S5, S6; Figures 3–5). Further, none of the few genes shared only by CM- and DCM-degrading strains among a small group of 5 *M. extorquens* strains were differentially expressed during growth with chlorinated methanes. Nevertheless looking for upregulated genes in CM/DCM-grown cultures vs. methanol-grown cultures will not detect constitutively expressed genes that support dehalogenation growth, and other complementary approaches need to be assessed (Ochsner et al., 2017). Among highly transcribed genes, a few had predicted functions in plasmid transfer and replication (genes *icmL, repA, repB, traD*; Figure S3). Thus, the part of the transcriptional response common to both chlorinated methanes was limited (Table S3). Adaptation to growth with chlorinated methanes seems to be largely a function of fine-tuning gene content/expression rather than large-scale changes as discussed in the following.

### Adaptive stress response

Indications for a transcriptional adaptive stress response to the utilization of chlorinated methanes in *M. extorquens* obtained here confirm previous suggestions from mutant growth phenotypes in the case of DCM (Muller et al., 2011b) and proteomic data in the case of CM (Roselli et al., 2013). Whereas expression of several genes associated with the general stress response was modulated (Tables S3, S5, S6; Figure 5), genes potentially associated with dehalogenation-specific stress were of particular interest. Since bacteria that grow with chlorinated methanes have to cope with production of intracellular hydrochloric acid production, one key question to address was which of intracellular proton or chloride buildup represents a larger stress for *M. extorquens*.

Intracellular generation of protons during growth with chlorinated methanes is expected to be detrimental for growth through intracellular acidification and also through dissipation of the proton-motive force and associated ATP production. It thus seems significant that the membrane-bound proton translocating pyrophosphatase *hppA* was one of only 12 genes of the core genome with higher expression on both chlorinated methanes. HppA-driven proton extrusion and restoration of the proton-motive force involves an additional energy expense through pyrophosphatase hydrolysis (Belogurov and Lahti, 2002).

Similarly, the H^+^/Cl^−^ antiporter ClcA involved in adaptation to chloride stress affords chloride efflux at a cost for the proton-motive force. The corresponding gene was expressed at high constitutive levels in strain DM4 (Figure S3B), as shown recently for DCM-degrading isolates including strain DM4 (Michener et al., 2014b). In case of strain CM4, *clcA* displayed moderate constitutive expression, but a pCMU01 plasmid-borne paralog *clcA2* (69% amino acid identity) was significantly more highly expressed in cultures on CM (Table S5; Figure S3A). This suggests that *clcA*-driven chloride extrusion is crucial for growth with both chlorinated methanes, and may involve different paralogs of this large gene family. Recent work showed that *clcA*, when transcribed from its native promoter cloned from strain DM4, confers higher fitness for growth with DCM but not with CM in *Methylobacterium* strains not previously exposed to chlorinated methanes (Michener et al., 2014a, 2016). It is noteworthy that the uncharacterized RND efflux transporter CliABC previously identified as chloride-induced in strain DM4 (Muller et al., 2011b) was found here to be even more expressed than gene *clcA* (Figure S3B). Taken together, the obtained data suggest that an increase in intracellular chloride levels represents more of the problem for growing strains of *M. extorquens* than intracellular proton production.

### Regulation under dehalogenative methylotrophic conditions

Strain-specific genes were among the most transcribed genes, especially those within gene clusters involved in chlorinated methane dehalogenation, i.e., *dcmA* and *cmuAB* genes (Figure 3). Here, CM-dependent transcription involved much larger changes in expression compared to DCM-dependent transcription (log_2_fc of 10 vs. 4, respectively). This confirms previous studies with RT-qPCR and transcriptional fusions of the *cmuA* promoter (Farhan Ul Haque et al., 2013). Among genes with increased expression levels with chlorinated methanes identified in this work, a significant number are annotated as putative regulatory genes (9 for CM and 6 for DCM respectively; Tables S5, S6). The transcriptional regulator of *dcmA* has been preliminarily characterized (La Roche and Leisinger, 1991; Muller et al., 2011b). In contrast, the genetic determinants involved in regulating the expression of the *cmu* pathway remain to be identified (Roselli et al., 2013).

The contribution of genes less expressed during growth with CM than with methanol was limited in the case of the core genome (11 genes) and even more so for the CM4 strain-specific genes (2 genes; Figure 5). This suggests that gene downregulation mediated by CM in strain CM4 is limited. On the other hand, a striking finding was that many plasmid pCMU01-borne paralogs but not their chromosomal homologs had higher transcript abundance in CM cultures (Figure 4B). This suggests the existence of as yet uncharacterized regulation mechanisms favoring expression of plasmid-encoded over chromosomally encoded gene paralogs in response to growth with CM.

### Effects of dehalogenation metabolism on transcription of methylotrophy genes

**Figure 6:**
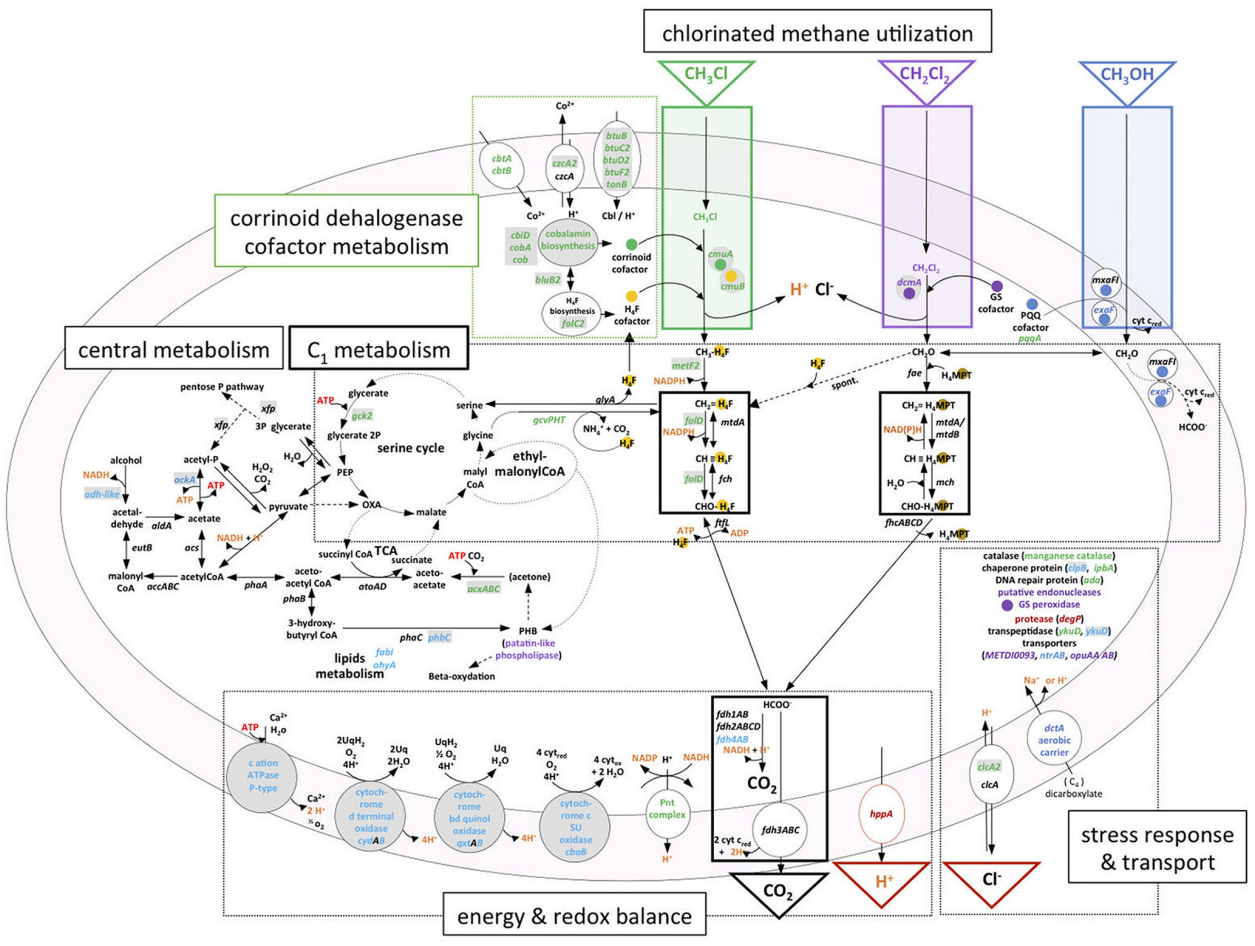
Transcriptional response and metabolism of chlorinated C_1_ compounds chloromethane and dichloromethane in *M. extorquens*. Chloromethane dehalogenation and methyl transfer to tetrahydrofolate (H_4_F) reactions are boxed in green; dichloromethane dehalogenation to formaldehyde in purple; methanol oxidation to formaldehyde in blue. Within C_1_ metabolism, black boxes delimit common methylotrophic modules (Chistoserdova et al., 2009): H_4_F and tetrahydromethanopterin (H_4_MPT)-dependent C_1_ transfer reactions, and formate oxidation for C_1_ dissimilation with C0_2_ production. Serine and ethylmalonyl-CoA cycles for C_1_ assimilation (Ochsner et al., 2015) are schematized with two connected circles. Reactions related to essential co-factors for chlorinated methane utilization are highlighted with colored spots; H_4_F (yellow), corrinoid cofactor (green), glutathione (purple), and pyrroquinoline quinone (blue). GEI-located genes in *M. extorquens* DM4 or in *M. extorquens* CM4 (plasmid pCMU01) are shown in gray-shaded boxes. RNA-Seq data are summarized using gene color-coding for more abundant transcripts on CM (green), DCM (purple), both CM and DCM (dark red), methanol [both *M. extorquens* CM4 and DM4 (dark blue), DM4 only (light blue)]. Compounds associated with energy-dependent transformations are highlighted in orange. Cbi, cobinamide; Cbl, cobalamin; CoA, coenzyme A; PEP, phosphoenolpyruvate; OXA, oxaloacetate; TCA, tricarboxylic acid cycle.

High levels of transcripts of genes *mxaFI* for subunits of the canonical methanol dehydrogenase (MDH) of *M. extorquens* (Amaratunga et al., 1997) were observed in methanol cultures, as expected, but also in cultures grown with chlorinated methanes (Figure 3). Since dehalogenation of DCM leads to formaldehyde, one explanation might be that transcription of methanol dehydrogenase MxaFI, which also converts formaldehyde (Nunn and Lidstrom, 1986), is also induced by formaldehyde. However, this does not strictly apply to growth with CM, as formaldehyde is not a direct product of CM dehalogenation by the *cmu* pathway (Vannelli et al., 1999; Figure 6) and chemical equilibrium between free formaldehyde and methylene-tetrahydrofolate (CH_2_ = H_4_F) does not favor free formaldehyde (Kallen and Jencks, 1966). This suggests that methanol dehydrogenase expression may be induced by other downstream metabolites common to CM and methanol catabolism, such as other folate-bound C_1_ compounds or formate, or that *mxaFI* expression is not switched off in strain CM4. On the other hand, other recently identified dehydrogenases active with methanol did not show the same transcriptional profile. In particular, the expression of genes for the XoxF-type enzymes was not modulated by chlorinated methanes. As for the PQQ-dependent ExaF-type dehydrogenase active with ethanol, methanol and formaldehyde (Good et al., 2016), seven genes directly upstream of gene *exaF* showed higher transcript levels in methanol-grown cultures in both CM4 and DM4 strains, including for gene *exaF* in strain CM4 (Table S5). Our results suggest complex modes of cross-regulation of key enzymes of methylotrophic metabolism in response to different C_1_ substrates that mostly involve genes that belong to the core genome (Figure 5).

### Metabolic fine-tuning for dehalogenative methylotrophic growth

Several genes of *M. extorquens* strains CM4 and DM4 that show significant modulation of expression with chlorinated methanes have predicted functions in energy homeostasis and redox processes. However, both the types of genes involved and the differential transcription patterns often differed between CM- and DCM-degrading strains. In the case of the DCM-degrading strain DM4, several gene clusters containing cytochrome oxidase genes displayed lower transcript counts in DCM cultures (Table S6). In particular, a substantial set of GEI-associated genes (Table 3), belonging to the variable genome of *M. extorquens* and potentially linked to cytochrome electron transport and thereby the transmembrane H^+^ gradient, had enhanced transcript abundance in cultures grown with methanol (Figure S4). Other potentially energy-associated adjustments of *M. extorquens* DM4 include very high transcript levels for various genes involved in metabolism of fatty acids and polyhydroxyalkanoates (Table S6), which are a storage form of carbon and energy (Escapa et al., 2012), suggesting that growth on DCM vs. methanol may involved different carbon and energy spillage control processes.

For the CM-degrading strain CM4, in contrast, obtained data suggest that energy balance adjustments as a function of growth with CM or methanol involve NAD(P)-linked metabolism. Whereas only one enzymatic step from methanol to formate involves one molecule of NAD(P)^+^ (MtdA/MtdB), for each molecule of CM transformed to formate, two molecules of NAD(P)H are potentially generated from NAD(P)^+^ in two successive reactions specifically catalyzed by MetF2 and FolD (Figure 6), These enzymes can use both NAD^+^ and NADP^+^ as cofactors, although *M. extorquens* CM4 FolD activity had only been assessed so far with NADP^+^ (Marx and Lidstrom, 2004). The corresponding genes *metF2* and *folD* are two of the most differentially expressed genes in the CM transcriptome (Table S5). Importantly, the *cmu* pathway specifically features FolD rather than MtdA and Fch for oxidation of C_1_ carbon to formate (Studer et al., 2002) unlike growth on methanol (Marx and Lidstrom, 2004). MetF2 and FolD may thus have different preferences than MtdA/MtdB for using NAD^+^ or NADP^+^ as cofactors. Should this be the case, significant altering of the intracellular pools of NAD^+^ and NADP^+^ during growth with CM compared to methanol could become growth limiting if levels of oxidized cofactors are limiting. A candidate for metabolic fine-tuning in this context is the membrane-bound transhydrogenase encoded by the chromosomal *pnt* gene cluster. All three *pnt* genes were significantly more expressed on CM at both transcription (Table S5) and protein (Roselli et al., 2013) levels. Transhydrogenase catalyzes the reversible reaction NADPH + NAD^+^ + ${\text{H}}_{\text{in}}^{+}$ <=> NADP^+^ + NADH + ${\text{H}}_{\text{out}}^{+}$ (Carroll et al., 2015). In addition to NADP^+^ regeneration via the membrane-bound transhydrogenase, concomitant proton efflux could thus potentially also help maintain internal cellular pH during CM dehalogenation.

During growth with CM, the high demand for oxidized NAD^+^/NADP^+^ cofactors may alter CH_2_ = H_4_F flux toward the serine cycle by limiting the carbon flux toward formate production. A compensatory metabolic rerouting of CH_2_ = H_4_F toward formate formation could be needed and may be achieved by the components of the so-called glycine cleavage complex (Figure 6), which were more highly abundant at both the transcript (GcvPHT, Table S5) and protein levels (GcvT; Roselli et al., 2013).

In conclusion, utilization of horizontally transferred genes for growth of *M. extorquens* with CM and DCM presumably involved required several post-transfer adjustments, as shown in recent experimental evolution experiments (Michener et al., 2014a, 2016). The new data obtained in the present study highlight potential global adjustments at the transcriptional level and more generally, the importance of substrate- and pathway-dependent genome adaptation following acquisition of new growth-supporting abilities such as degradation of toxic halogenated compounds. Clearly, *M. extorquens* represents a model of choice to address these issues in the future.

## Author contributions

PC, BM, and FB prepared RNA. PC and AA constructed cDNA libraries. PC and FB performed RNA-Seq analysis. BM carried out RT-qPCR assays and data processing. FB designed the study. PC, MW, SV, FB, and SC participated in bioinformatic analysis. PC, BM, SK, SV, SC, and FB were involved in data analysis and interpretation. PC, SV, and FB wrote the manuscript. All authors read and approved the final manuscript.

### Conflict of interest statement

The authors declare that the research was conducted in the absence of any commercial or financial relationships that could be construed as a potential conflict of interest.

## Abbreviations

CM: chloromethane
DCM: dichloromethane
GEI: genomic island
H_4_F: tetrahydrofolate.
