# Supplementary material for: Genomic and Transcriptomic Analysis of Growth-Supporting Dehalogenation of Chlorinated Methanes in Methylobacterium
Source: Front Microbiol. 2017 Sep 1;8:1600. doi: 10.3389/fmicb.2017.01600 (PMC5585157; doi:10.3389/fmicb.2017.01600)
Supplement: Supplementary file 1 [file DataSheet1.doc]

Supplementary Material

Genome content and expression for growth-supporting dehalogenation

in Methylobacterium extorquens

Pauline Chaignaud, Bruno Maucourt, Marion Weiman, Adriana Alberti, Steffen Kolb, Stéphane Cruveiller, Stéphane Vuilleumier, Françoise Bringel^*^

*** Correspondence:** Françoise Bringel, francoise.bringel@unistra.fr

# Supplementary Figures

# Supplementary Tables

# References


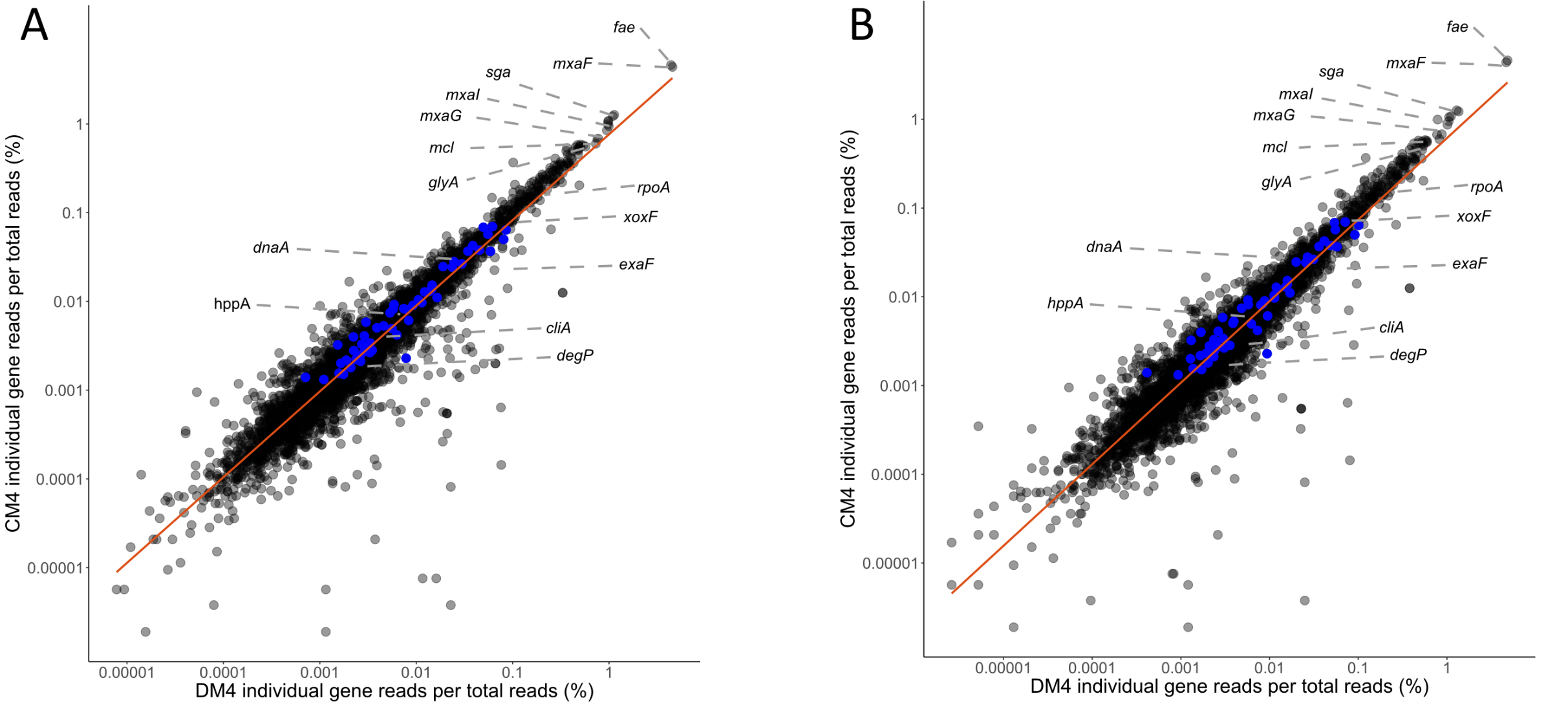


**Supplementary Figure S1.** **Comparison of genome-wide gene transcription of *M. extorquens* strains CM4 and DM4 grown with methanol**. Read percentage represents the number of reads for each gene divided by the cumulated number of reads of the 4,620 genes shared by the 2 strains (at least 80% of identity on the protein level on 80% of the CDS length). Names of some highly transcribed genes are indicated. Genes shared by the strains CM4 and DM4 but not by other strains of *M. extorquens* are indicated in blue (list in Table S3). (A) Normalisation using 55 reference genes with unchanged log_2_fc (Table S2). (B) Normalisation using the complete set of CDS by the standard method (Anders et al., 2013).


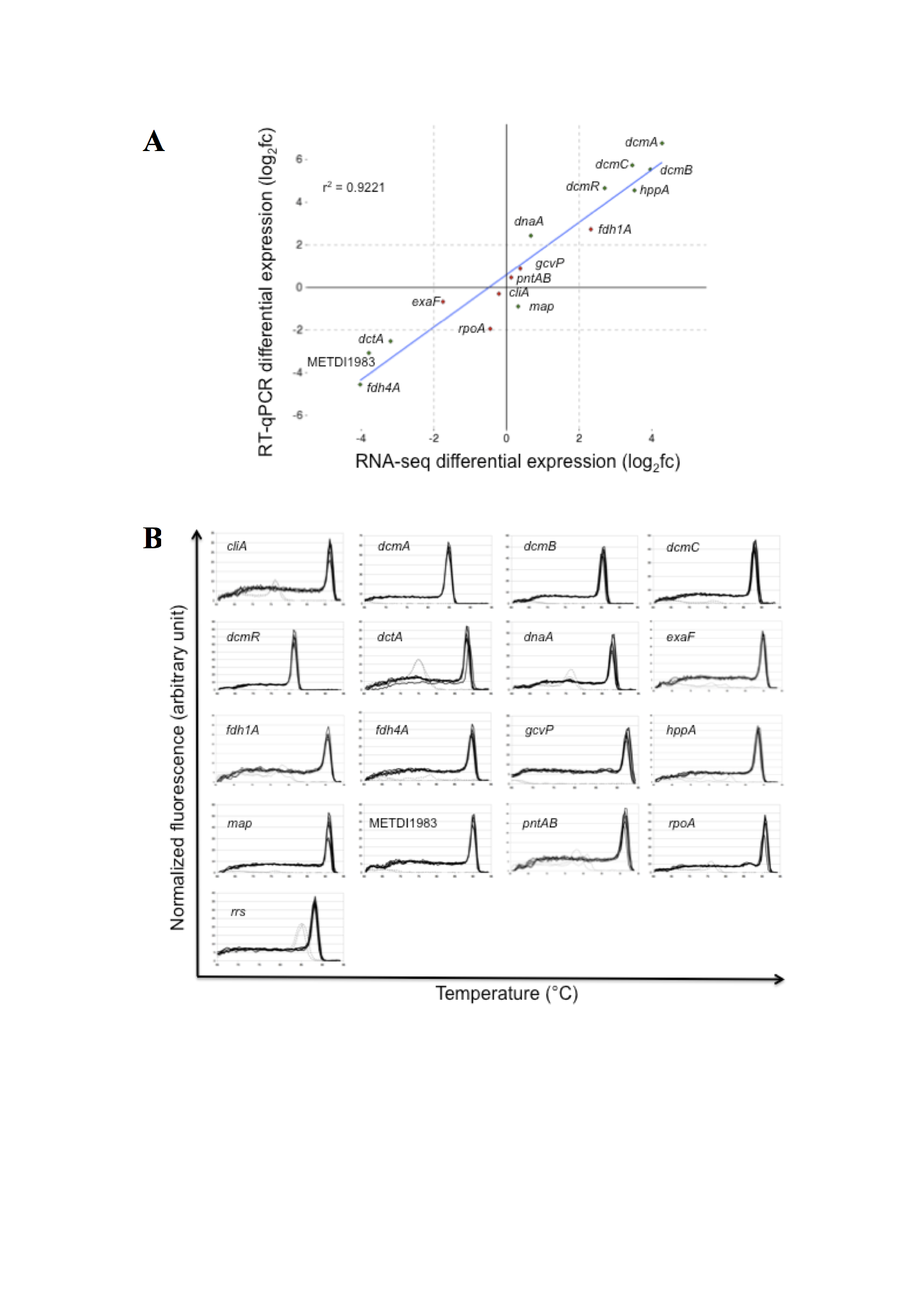


**Supplementary Figure S2.** **Validation of RNA-Seq results by RT-qPCR.** (A) Gene expression of 16 genes compared in independent replicate cultures by RNA-Seq (X axis) and RT-qPCR (Y axis). Genes indicated in green or red have a *p*-value ≤ 0.1 or ≥ 0.1 respectively in the RNA-Seq experiment. (B) Melting curves for replicate qPCR reactions for each of the 17 genes. A single peak was observed with *M. extorquens* DM4 gDNA (black line), while no amplification was detected in the absence of DNA template (grey line).

**A**

**
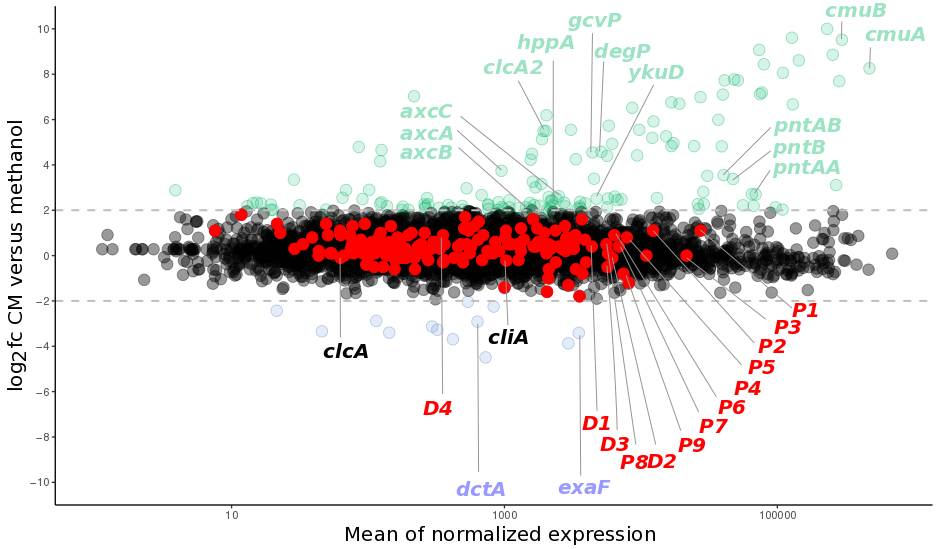
**

**B**

**
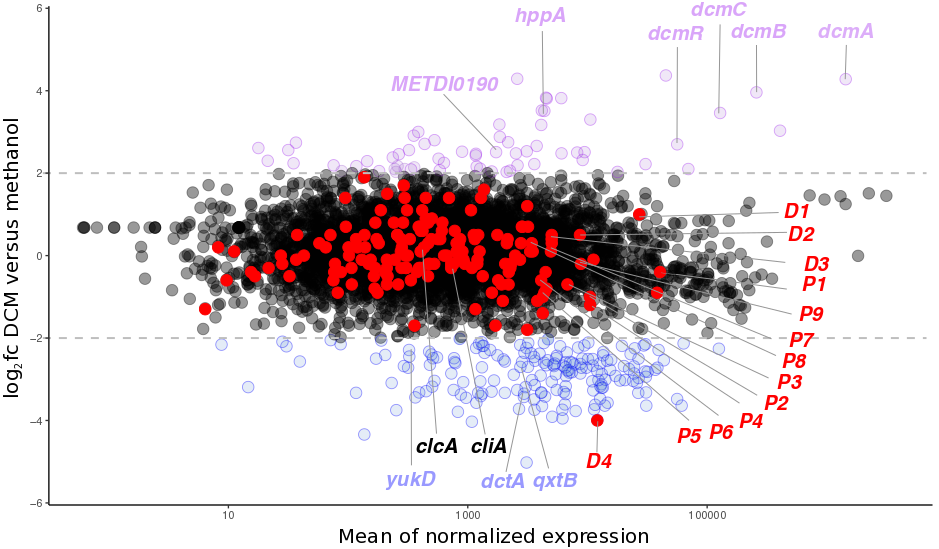
**

**Supplementary Figure S3.** **MA-plot representation of the transcript abundance**. The abscissa scales logarithmically the mean of normalized read numbers divided by the kb of gene length between the tested growth conditions. The ordinate scales logarithmically the log_2_ of the fold change of normalized reads in growth with chlorinated methanes compared to with methanol. Genes considered as not differentially abundant have log_2_fc between 2 and -2. Genes more abundant in cells grow with chloromethane or dichloromethane compared to methanol are indicated in green and pink, respectively. Genes more abundant in cells grow with methanol are indicated in blue. The 163 genes shared only by the dechlorinating strains *M. extorquens* CM4 and DM4 are indicated in red color and include plasmid- and chromosome-borne genes that are names P and D, respectively: P1, Mchl_5591/p1METDI0125; P2, Mchl_5600/p1METDI0134; P3, Mchl_5607/ 1METDI0141; P4, *icmL*; P5, Mchl_5605/p1METDI0139; P6, Mchl_5606/p1METDI0140; P7, *repA*; P8, *repB*; P9, *traD*; D1, *osmB*-like; D2, Mchl_4205/METDI0774; D3, Mchl_4551/METDI5181, and D4, Mchl_4141/METDI4814. METDI0190 encode for a putative glutathione peroxidase of the core genome. (A) CM versus methanol for *M. extorquens* CM4; (B) DCM versus methanol for *M. extorquens* DM4.

#
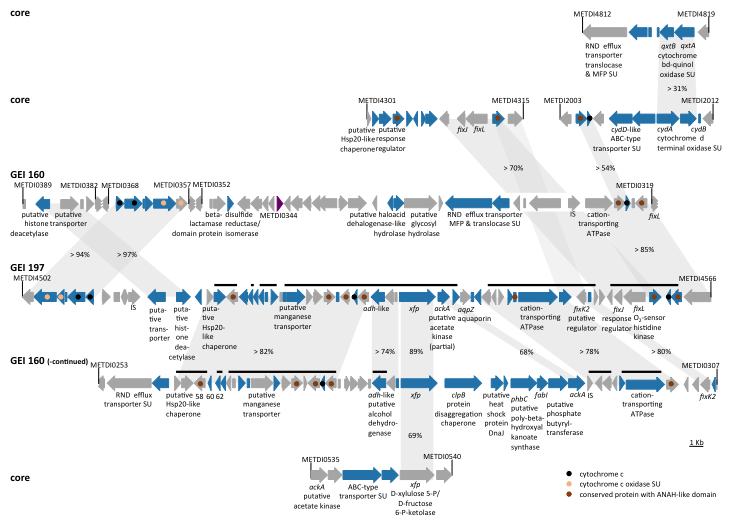


**Supplementary Figure S4. Gene content and transcript abundance similarities between two GEIs in *M. extorquens* DM4**. Conserved stretches between two GEI-borne regions (Table 3) are delimited with grey rectangles. Numbers indicate the percentage of amino acid identity between encoded METDI protein labels defined in MicroScope (https://www.genoscope.cns.fr/agc/microscope/mage/viewer. php?S_id=240). Genes are indicated as colored arrows: in blue when higher normalized read numbers were detected in cultures grown with methanol compared to with dichloromethane (log_2_fc ≤ -2), in grey when no significant difference was observed (-2 < log_2_fc values >2), and in purple for the METDI0344-encoding gene detected with higher reads in dichloromethane (log_2_fc value of 2.2). Interrupted GEI segments are indicated with zigzagged arrows. The black lines indicate GEI partially conserved in the *M. extorquens* AM1 strain. IS stands for insertion sequences. The ANAH conserved domain is found in proteins of the “adenine nucleotide alpha hydrolases-like superfamily” including N type ATP PPases, ATP sulphurylases, universal stress response proteins and electron transfer flavoproteins (http://supfam.cs.bris.ac.uk/SUPERFAMILY/cgi-bin/scop.cgi?sunid=52402).

## Supplementary Tables

**Table S1.** List of primers

| **Gene** | **Product** | **Primer set and 5’->3’ sequence ^a^** | **Size (bp)** | ***M. extorquens* genome localization ^b^** | |  |
| --- | --- | --- | --- | --- | --- | --- |
|  |  |  |  | **strain CM4** | **strain DM4** |  |
| **For PCR validation of DNAse-treated RNAs** | | | | | | |
| *rrs* | 16S rRNA | 27f GTTTGATCCTGGCTCAG 927r CCGTCAATTCCTTT**R**AGTTT | 859 | 5 copies (100% Id) | 5 copies (100% Id) |  |
| *cmuA* | CH_3_Cl methyltransferase /corrinoid-binding protein | cmuA802F TTCAACGGCGA**Y**ATGTATCC**Y**GG cmuA1244R TA**B**TCCAT**D**ATGGC**Y**TCGA | 442 | 336191-336213 335790-335771 | not detected |  |
| *dcmA* | dichloromethane dehalogenase | dcmAiF CGATCTAGATAGCGCGGTCTAAGCGACT dcmAendR TCGATCTAGATAGCGCGGTCTAAGCGAC | 879 | not detected | 2562957-2562974 2563815-2563834 |  |
| **For RT qPCR validation of RNAseq data** | | | | | | |
| *cliA* | RND efflux transporter, MFP subunit | cliA1f ATCGTCCTGAGTCGCACCTT cliA1r CGTGACGTGGATCGTCTTGT | 153 | 4994842-4994861 4994709-4994728 | 5279398-5279417 5279265-5279284 |  |
| *dcmA* | dichloromethane dehalogenase | TnA1f TTCGGCTGTTTGATCTATGG TnA1r ATCCGCATAGCTGACCTCAT | 159 | nd | 2563344-2563363 2563484-2563502 |  |
| *dcmB* | conserved protein of unknown function DcmB | DcmCf0 GCGACGCATCTTACTTCGAT TnB1f AGGGCTTGCTCAATCAAAT | 150 | nd | 2564955-2564974 264825-2564844 |  |
| *dcmC* | conserved protein of unknown function DcmC | TnCf CGGCGTCTGGTCATTTGG DcmCr ATATCACTGCCCAGGTGTCG | 152 | nd | 2565225-2565242 2565357-2565376 |  |
| *dcmR* | transcriptional regulator of DCM dehalogenase | TnR1f AGGTGCACAAAGCCATTAAA TnR1r GCCCACTCCATATCACCAAC | 151 | nd | 2561793-2561812 2561662-2561681 |  |
| *dctA* | C4-dicarboxylate transport protein | dctAf TGCATATCGACCCGAACTC dctAr GAAGAACAGCACCTGGAGGA | 155 | 3460114-3460132  3459978-3459997 | 3765633-3765651 3765497-3765516 |  |
| *dnaA* | DNA replication initiator protein | dnaAf CCGCCGGGTGATCTATCTCA dnaAr GTCTGGATCGACTTGCCCTG | 155 | 2109-2128 2245-2264 | 1381-1400 1517-1536 |  |
| *exaF* | quinoprotein methanol/ ethanol dehydrogenase | mdh2f GCTCCTATTCGCGCCTCTAC  mdh2r TGCCGAAATAGACCTTGTCC | 147 | 1595980-1595999  1596107-1596126 | 1859529- 1859548  1859656- 1859675 |  |
| *fdh1A* | tungsten-containing formate dehydrogenase alpha subunit | fdh1Af CCAAGGTCGTGTTCGACTTC fdh1Ar ATAGACCGTCCGGGTCTGG | 137 | 5391775-5391794  5391893-5391911 | 5604120- 5604139  5604238- 5604256 |  |
| *fdh4A* | formate dehydrogenase subunit A | fdh4Af CTGCGAACTCACCGTCCAC  fdh4Ar ACCATGCTCATCGAATCCTC | 156 | 2522699-2522717  2522559-2522578 | 2809514- 2809532  2809374- 2809393 |  |
| *gcvP* | glycine cleavage complex protein P | gcvPf1 CCTCGATGTTCGCGGTCTAT gcvPr1 ACGGTGATCGTGTCGAAGAA | 145 | 831525-831544  831400-831419 | 911108-911127 910983-911002 |  |
| *hppA* | H^+^ translocating pyrophosphate synthase | hppAf GCTCGAAGC**S**ATGATGATCT hppAr ATCAGGCCCTTGTAGAGTGC | 129 | 3593391-3593410  3593282-3593301 | 3798512-3798531 3798403- 3798422 |  |
| *map* | methionine aminopeptidase | mapf AACCTCGATTGCTGCCTGAT mapR GGATTC**S**GC**R**TAAG**W**CTGGA | 198 | | 2608953-2608973 2609127-2609147 | | --- | | 2898334-2898354 2898508-2898528 |  |
| - | METDI1983, ExaF-associated cytochrome c550 | mdh3f GCGCGAGGAGAATCCTTAT  mdh3r GTACTCGTCGCCCTCCTTG | 160 | 1594210-1594228  1594351-1594369 | 1857771-1857789  1857912-1857930 |  |
| *pntAB* | NAD(P)^+^ transhydrogenase, subunit alpha part 2 | pntABf TGACATCGCCCATCAGAAC  pntABr ACGACGTAGTAGCCGACGAA | 150 | 3151719-3151737  3151849-3151868 | 3457018-3457036  3457148-3457167 |  |
| *rpoA* | DNA-directed RNA polymerase alpha chain | RpoAf TCGTCCTCAACATCAAGACCA RpoAr GAGGTCGGGGTTCAGGATCT | 143 | 2590269-2590289  2590147-2590166 | 2877354-2877374  2877232-2877251 |  |
| *rrs* | 16S rRNA | Mex1369f CGGTGAATACGTTCCCGG Mex1492r GGCTACCTTGTTACGACTT | 138 | 5 copies (100% Id)) | 5 copies (100% Id) |  |

^a^ In bold, degenerate nucleotides with B= C, G or T ; D =; A, G or T; S = G or C; R = A or G; y= c or T ; W = A or T.

^b^ Chromosome positions are given as in MicroScope (<https://www.genoscope.cns.fr/agc/microscope/mage/viewer.php>).

**Table S2. Genes used for RNAseq data normalization**

| **Label^a^** | | **Gene** | **Product** |
| --- | --- | --- | --- |
| **Strain CM4** | **Strain DM4** |
| Mchl_0002 | METDI0001 | *dnaA* | DNA replication initiator protein |
| Mchl_0220 | METDI4779 | *-* | putative phosphodiesterase |
| Mchl_0372 | METDI0202 | *glnE* | glutamate-ammonia-ligase adenylyltransferase |
| Mchl_0480 | METDI0517 | *zwf* | glucose-6-phosphate 1-dehydrogenase |
| Mchl_0641 | METDI0609 | *-* | conserved protein of unknown function |
| Mchl_0710 | METDI0680 | *-* | conserved protein of unknown function |
| Mchl_0713 | METDI0683 | *qxtB* | cytochrome bd ubiquinol oxidase, subunit II |
| Mchl_0917 | METDI1100 | *prmC* | protein-(glutamine-N5) methyltransferase |
| Mchl_1568 | METDI2018 | *-* | putative CelB-like protein |
| Mchl_1668 | METDI2051 | *-* | conserved protein of unknown function precursor |
| Mchl_1859 | METDI2245 | *trmE* | tRNA modification GTPase trmE |
| Mchl_2123 | METDI2469 | *lysA* | diaminopimelate decarboxylase |
| Mchl_2201 | METDI2686 | *-* | conserved protein of unknown function |
| Mchl_2204 | METDI2689 | *-* | transglutaminase family protein cysteine peptidase |
| Mchl_2251 | METDI2738 | *-* | putative DNA topoisomerase I |
| Mchl_2277 | METDI2766 | *gmk* | guanylate kinase |
| Mchl_2458 | METDI2953 | *adk* | adenylate kinase |
| Mchl_2482 | METDI2981 | *map* | methionine aminopeptidase |
| Mchl_2549 | METDI3056 | *rnc* | ribonuclease III |
| Mchl_2552 | METDI3059 | *rsuA* | 16S rRNA pseudouridylate 516 synthase |
| Mchl_2581 | METDI3088 | *ung* | uracil-DNA glycosylase superfamily protein |
| Mchl_2691 | METDI3197 | *-* | ribonuclease BN family protein |
| Mchl_2750 | METDI3257 | *-* | putative decarboxylase, thiamine pyrophosphate requiring |
| Mchl_2817 | METDI3327 | *thrB* | homoserine kinase |
| Mchl_2824 | METDI3334 | *recA* | RecA protein |
| Mchl_3177 | METDI3720 | *lpxC* | UDP-3-O-acyl N-acetylglucosamine deacetylase |
| Mchl_3288 | METDI3840 | *gatB* | aspartyl/glutamyl-tRNA amidotransferase subunit B |
| Mchl_3478 | METDI3942 | *-* | ATP-dependent DNA helicase |
| Mchl_3479 | METDI3943 | *-* | conserved protein of unknown function |
| Mchl_3486 | METDI3950 | *ribE* | 6,7-dimethyl-8-ribityllumazine synthase |
| Mchl_3504 | METDI3967 | *mutY* | A/G-specific adenine glycosylase |
| Mchl_3512 | METDI3975 | *-* | putative adenylate cyclase protein |
| Mchl_3524 | METDI3989 | *rimI* | ribosomal-protein-alanine N-acetyltransferase |
| Mchl_3541 | METDI4005 | *murI* | glutamate racemase |
| Mchl_3578 | METDI4045 | *thrC* | threonine synthase |
| Mchl_3610 | METDI4078 | *nhaA* | sodium: proton antiporter |
| Mchl_3635 | METDI4104 | *pyrB* | aspartate carbamoyltransferase |
| Mchl_3693 | METDI4186 | *-* | conserved protein of unknown function |
| Mchl_3946 | METDI4648 | *fadD* | acyl-CoA synthetase |
| Mchl_4052 | METDI4721 | *-* | tonB-dependent siderophore receptor protein |
| Mchl_4185 | METDI4861 | *prs* | phosphoribosylpyrophosphate synthetase |
| Mchl_4253 | METDI4871 | *gid* | tRNA uridine 5-carboxymethylaminomethyl modification enzyme |
| Mchl_4306 | METDI4930 | *dnaB* | replicative DNA helicase DnaB |
| Mchl_4344 | METDI4968 | *-* | putative cysteine desulfurase pyridoxal P-dependent |
| Mchl_4404 | METDI5028 | *ftsK* | cell division protein |
| Mchl_4487 | METDI5112 | *-* | conserved protein of unknown function |
| Mchl_4851 | METDI5422 | *prfC* | peptide chain release factor RF-3,GTP-binding factor |
| Mchl_4855 | METDI5427 | *tuf* | elongation factor Tu (EF-Tu) |
| Mchl_4914 | METDI5486 | *-* | putative ATP-independent RNA helicase |
| Mchl_5105 | METDI5692 | *dnlJ* | DNA ligase, NAD(^+^)-dependent |
| Mchl_5143 | METDI5731 | *lpxB* | lipid-A-disaccharide synthase |
| Mchl_5159 | METDI5748 | *valS* | valine tRNA synthetase |
| Mchl_5243 | METDI5833 | *pbpC* | penicillin-binding protein |
| Mchl_5271 | METDI5861 | *hemA* | 5-aminolevulinic acid synthase |
| Mchl_5315 | METDI5905 | *-* | conserved protein of unknown function |

^a^ MaGe annotation (<https://www.genoscope.cns.fr/agc/microscope>) of 55 genes with constitutive transcript abundance in the tested condition (log_2_fc value between -0.9 and 1.2 in cultures grown with chlorinated methanes compared to with methanol).

**Table S3.** Variable genome shared only by dehalogenating *M. extorquens* strains CM4 and DM4

| **Label ^a^** | | **b** | **Gene** | **Function** |
| --- | --- | --- | --- | --- |
| **Strain CM4** | **Strain DM4** |
| Mchl_0185 | METDI0124 |  | *-* | protein of unknown function |
| MCv2_0417 | METDI0198 |  | *-* | protein of unknown function |
| Mchl_3333 | METDI0326 |  | *-* | putative sensory box-containing diguanylate cyclase/phosphodiesterase, PAS/PAC, GGDEF and EAL domains |
| Mchl_0688 | METDI0657 |  | *-* | putative flagellin protein FlaA |
| Mchl_4205 | METDI0774 | 1 | *-* | protein of unknown function |
| Mchl_4206 | METDI0775 | 1 | *sucD* | succinyl-CoA synthetase, alpha subunit |
| Mchl_4207 | METDI0776 | 1 | *sucC* | succinyl-CoA synthetase, beta subunit |
| Mchl_4208 | METDI0777 | 1 | *-* | putative succinate-semialdehyde dehydrogenase |
| Mchl_4209 | METDI0778 | 1 | *-* | putative transporter, major facilitator superfamily |
| Mchl_4211 | METDI0781 | 1 | *-* | glucose-methanol-choline oxidoreductase |
| Mchl_4212 | METDI0782 | 1 | *-* | MFS transporter membrane protein |
| Mchl_4214 | METDI0788 | 1 | *xsc* | sulfoacetaldehyde acetyltransferase |
| Mchl_4215 | METDI0789 | 1 | *-* | DNA-binding transcriptional repressor, IclR family |
| Mchl_4216 | METDI0790 | 1 | *-* | putative acetate kinase (AckA) |
| MCv2_0842 | METDI0940 |  | *-* | protein of unknown function |
| Mchl_0906 | METDI1089 |  | *-* | putative OsmB-like lipoprotein |
| MCv2_1071 | METDI1130 |  | *-* | protein of unknown function |
| Mchl_0969 | METDI1157 | 2 | *-* | putative site-specific recombinase |
| Mchl_0970 | METDI1158 | 2 | *-* | putative site-specific recombinase |
| Mchl_0971 | METDI1159 | 2 | *-* | conserved protein of unknown function |
| MCv2_1101 | METDI1161 | 2 | *-* | protein of unknown function |
| Mchl_0974 | METDI1162 | 2 | *-* | putative ferrichrome-iron receptor protein |
| Mchl_0975 | METDI1163 | 2 | *-* | putative exported esterase (LroE-like) |
| Mchl_0976 | METDI1164 | 2 | *-* | protein of unknown function |
| Mchl_0977 | METDI1165 | 2 | *-* | protein of unknown function |
| Mchl_0979 | METDI1168 | 2 | *-* | putative resolvase/recombinase |
| Mchl_0980 | METDI1170 | 2 | *-* | putative resolvase/recombinase |
| MCv2_1114 | METDI1171 | 2 | *-* | conserved protein of unknown function |
| MCv2_1115 | METDI1172 | 2 | *-* | protein of unknown function |
| Mchl_0982 | METDI1174 | 2 | *-* | putative phosphohydrolase/phosphoesterase |
| Mchl_0983 | METDI1175 | 2 | *-* | putative transcriptional repressor |
| MCv2_1120 | METDI1179 | 2 | *-* | protein of unknown function |
| Mchl_0984 | METDI1180 | 2 | *-* | protein of unknown function |
| Mchl_0985 | METDI1181 | 2 | *-* | putative ATP-hydrolyzing enzyme |
| MCv2_1217 | METDI1202 |  | *-* | putative exported protein of unknown function |
| MCv2_1218 | METDI1203 |  | *-* | putative exported protein of unknown function |
| Mchl_1080 | METDI1216 |  | *-* | putative exported protein of unknown function |
| Mchl_4729 | METDI1222 |  | *-* | putative exported protein of unknown function |
| Mchl_1114 | METDI1282 |  | *-* | conserved exported protein of unknown function |
| Mchl_0988 | METDI1314 |  | *-* | fragment of protein of unknown function (part 2) |
| Mchl_0990 | METDI1315 |  | *-* | protein of unknown function |
| MCv2_1140 | METDI1329 |  | *-* | protein of unknown function |
| Mchl_1005 | METDI1336 |  | *-* | putative exported protein of unknown function |
| MCv2_1149 | METDI1339 |  | *-* | protein of unknown function |
| Mchl_1022 | METDI1354 |  | *-* | protein of unknown function |
| Mchl_1037 | METDI1369 |  | *-* | protein of unknown function |
| Mchl_1136 | METDI1461 | 3 | *-* | permease of the major facilitator superfamily (MFS_1) |
| Mchl_1137 | METDI1462 | 3 | *-* | short-chain dehydrogenase/reductase SDR |
| Mchl_1138 | METDI1463 | 3 | *-* | conserved exported protein of unknown function |
| Mchl_1141 | METDI1467 | 3 | *-* | putative short-chain dehydrogenase/reductase SDR |
| Mchl_1142 | METDI1468 | 3 | *-* | putative transcriptional regulator, LysR family |
| Mchl_1143 | METDI1469 | 3 | *-* | oxidoreductase, 2Fe-2S subunit |
| Mchl_1144 | METDI1470 | 3 | *-* | oxidoreductase, FAD-binding subunit |
| Mchl_1145 | METDI1471 | 3 | *-* | oxidoreductase, molybdopterin-binding subunit |
| Mchl_1147 | METDI1473 | 3 | *-* | protein of unknown function |
| MCv2_1316 | METDI1474 | 3 | *-* | putative membrane protein of unknown function |
| Mchl_1259 | METDI1666 | 4 | *-* | putative transcriptional regulator, LysR family |
| Mchl_1260 | METDI1667 | 4 | *-* | ABC transporter, periplasmic protein; putative taurine transporter |
| Mchl_1261 | METDI1668 | 4 | *-* | ABC transporter, permease component; putative taurine transporter |
| Mchl_1262 | METDI1669 | 4 | *-* | ABC transporter, ATPase; putative taurine transporter |
| Mchl_1264 | METDI1671 | 4 | *-* | glutamyl-tRNA(Gln) amidotransferase subunit A |
| MCv2_1584 | METDI1797 |  | *-* | conserved protein of unknown function |
| MCv2_1680 | METDI1888 |  | *-* | protein of unknown function |
| Mchl_5331 | METDI1912 |  | *-* | protein of unknown function |
| MCv2_1703 | METDI1921 | 5 | *-* | putative phosphoglycerate/bisphosphoglycerate mutase |
| Mchl_1480 | METDI1923 | 5 | *-* | metal-dependent phosphohydrolase, HD subdomain |
| Mchl_1481 | METDI1924 | 5 | *-* | transcriptional regulator, AraC family |
| Mchl_1482 | METDI1925 | 5 | *-* | protein of unknown function; putative membrane protein |
| Mchl_1484 | METDI1927 | 5 | *-* | protein of unknown function |
| Mchl_1493 | METDI1935 | 5 | *-* | conserved protein of unknown function |
| MCv2_1739 | METDI1950 | 5 | *-* | protein of unknown function |
| MCv2_1740 | METDI1951 | 5 | *-* | putative membrane protein of unknown function |
| MCv2_1741 | METDI1952 | 5 | *-* | conserved protein of unknown function |
| MCv2_1751 | METDI1961 | 5 | *-* | conserved exported protein of unknown function |
| MCv2_2296 | METDI2356 |  | *-* | conserved protein of unknown function |
| MCv2_2310 | METDI2369 |  | *-* | protein of unknown function |
| Mchl_5330 | METDI2680 |  | *-* | putative SAM-dependent methyltransferase domain |
| Mchl_2249 | METDI2736 |  | *-* | protein of unknown function |
| MCv2_2529 | METDI2759 |  | *-* | protein of unknown function |
| Mchl_2370 | METDI2862 |  | *-* | protein of unknown function |
| MCv2_2712 | METDI2882 |  | *-* | protein of unknown function |
| MCv2_2972 | METDI3136 |  | *-* | protein of unknown function |
| MCv2_3197 | METDI3358 |  | *-* | protein of unknown function |
| Mchl_2863 | METDI3394 |  | *-* | protein of unknown function |
| MCv2_3231 | METDI3407 |  | *-* | conserved protein of unknown function |
| MCv2_3537 | METDI3739 |  | *-* | protein of unknown function |
| Mchl_3241 | METDI3788 |  | *-* | conserved protein of unknown function |
| Mchl_3438 | METDI3901 |  | *-* | conserved protein of unknown function |
| Mchl_3466 | METDI3928 |  | *-* | putative addiction module antidote protein |
| Mchl_0111 | METDI4323 |  | *-* | protein of unknown function |
| Mchl_0318 | METDI4325 |  | *-* | protein of unknown function |
| Mchl_3840 | METDI4356 |  | *-* | fragment of phage integrase (part 1) |
| MCv2_5403 | METDI4380 |  | *-* | conserved protein of unknown function |
| Mchl_3913 | METDI4612 |  | *-* | conserved protein of unknown function |
| Mchl_0225 | METDI4784 |  | *-* | putative exported protein of unknown function |
| Mchl_4141 | METDI4814 |  | *-* | putative exported protein of unknown function |
| MCv2_4813 | METDI4912 |  | *-* | protein of unknown function |
| Mchl_4341 | METDI4965 |  | *-* | conserved protein of unknown function |
| Mchl_4342 | METDI4966 |  | *-* | conserved protein of unknown function |
| Mchl_4350 | METDI4974 |  | *-* | conserved protein of unknown function |
| MCv2_4420 | METDI5044 |  | *-* | protein of unknown function |
| Mchl_4444 | METDI5071 |  | *-* | putative exported protein of unknown function |
| Mchl_4550 | METDI5180 |  | *-* | conserved protein of unknown function |
| Mchl_4551 | METDI5181 |  | *-* | conserved protein of unknown function |
| Mchl_4659 | METDI5293 |  | *-* | conserved protein of unknown function |
| Mchl_4718 | METDI5356 | 6 | *-* | putative phage integrase |
| Mchl_4719 | METDI5357 | 6 | *-* | putative transcriptional regulator |
| Mchl_4720 | METDI5358 | 6 | *-* | putative metallo-beta-lactamase family protein |
| Mchl_4721 | METDI5359 | 6 | *-* | conserved protein of unknown function |
| Mchl_4722 | METDI5360 | 6 | *-* | putative exported or membrane protein of unknown function |
| Mchl_4723 | METDI5361 | 6 | *-* | protein of unknown function |
| Mchl_4742 | METDI5367 | 6 | *-* | protein of unknown function |
| Mchl_4743 | METDI5371 | 6 | *-* | protein of unknown function |
| Mchl_4744 | METDI5372 | 6 | *-* | putative DNA helicase related protein |
| Mchl_4749 | METDI5375 | 6 | *-* | putative ardC antirestriction protein |
| MCv2_5307 | METDI5397 |  | *-* | protein of unknown function |
| Mchl_5447 | METDI0315  METDI0325 |  | *-* | transposase |
| Mchl_5456 | METDI4486 | 7 | *-* | putative membrane protein of unknown function |
| Mchl_5457 | METDI4485 | 7 | *ispF* | 2C-methyl-D-erythritol 2,4-cyclodiphosphate synthase |
| Mchl_5458 | METDI4484 | 7 | *-* | putative pseudouridine synthase, putative 1-deoxy-D-xylulose 5-phosphate reductoisomerase |
| Mchl_5459 | METDI4483 | 7 | *gdhA* | glutamate dehydrogenase (NAD(P)+) oxidoreductase protein |
| Mchl_5461 | METDI4481 | 7 | *-* | putative oxidoreductase |
| Mchl_5546 | p1METDI0092 | 8 | *-* | conserved protein of unknown function |
| Mchl_5549 | p1METDI0095 | 8 | *-* | protein of unknown function |
| Mchl_5552 | p1METDI0098 | 8 | *-* | conserved protein of unknown function |
| Mchl_5555 | p1METDI0102 | 8 | *-* | conserved protein of unknown function |
| Mchl_5558 | p1METDI0104 | 8 | *ardC* | antirestriction protein ArdC |
| Mchl_5559 | p1METDI0105 | 8 | *-* | conserved protein of unknown function |
| Mchl_5564 | p1METDI0110 | 8 | *-* | conserved protein of unknown function |
| Mchl_5567 | p1METDI0111 | 8 | *-* | putative methylase/helicase |
| Mchl_5568 | p1METDI0112 | 8 | *-* | conserved protein of unknown function |
| Mchl_5569 | p1METDI0113 | 8 | *-* | conserved protein of unknown function |
| Mchl_5571 | p1METDI0117 | 8 | *-* | protein of unknown function |
| Mchl_5572 | p1METDI0118 | 8 | *traG* | conjugal transfer protein |
| Mchl_5573 | p1METDI0119 | 8 | *traD* | conjugal transfer protein |
| Mchl_5575 | p1METDI0121 | 8 | *-* | putative conjugal transfer protein (TraA) |
| Mchl_5578 | p1METDI0122 | 8 | *-* | protein of unknown function |
| Mchl_5579 | p1METDI0123 | 8 | *-* | protein of unknown function |
| Mchl_5591 | p1METDI0125 | 8 | *-* | conserved protein of unknown function; putative membrane protein |
| Mchl_5592 | p1METDI0126 | 8 | *icmB* | conserved protein of unknown function; IcmB/DotO-related protein |
| Mchl_5595 | p1METDI0129 | 8 | *icmE* | putative IcmE-related protein |
| Mchl_5596 | p1METDI0130 | 8 | *icmK* | putative IcmK-related protein |
| Mchl_5597 | p1METDI0131 | 8 | *icmL* | conserved protein of unknown function; putative IcmL (DotI) protein |
| Mchl_5600 | p1METDI0134 | 8 | *-* | putative membrane protein of unknown function |
| Mchl_5601 | p1METDI0135 | 8 | *-* | protein of unknown function |
| Mchl_5602 | p1METDI0136 | 8 | *-* | putative DotC-related protein |
| Mchl_5603 | p1METDI0137 | 8 | *-* | putative Dot/Icm secretion system ATPase DotB |
| Mchl_5604 | p1METDI0138 | 8 | *-* | conserved protein of unknown function, putative exported protein |
| Mchl_5605 | p1METDI0139 | 8 | *-* | protein of unknown function |
| Mchl_5606 | p1METDI0140 | 8 | *-* | conserved protein of unknown function; putative exported protein |
| Mchl_5607 | p1METDI0141 | 8 | *-* | putative exported protein of unknown function |
| Mchl_5609 | p1METDI0002 | 8 | *-* | conserved protein of unknown function |
| Mchl_5610 | p1METDI0003 | 8 | *-* | conserved exported protein of unknown function |
| Mchl_5611 | p1METDI0004 | 8 | *-* | of unknown function |
| Mchl_5612 | p1METDI0005 | 8 | *-* | putative membrane protein of unknown function |
| Mchl_5613 | p1METDI0006 | 8 | *-* | protein of unknown function; putative exported or membrane protein |
| Mchl_5614 | p1METDI0007 | 8 | *-* | fragment of transposase related to IS481 family |
| Mchl_5615 | p1METDI0008 | 8 | *repA* | plasmid partitioning protein RepA |
| Mchl_5616 | p1METDI0009 | 8 | *repB* | plasmid partitioning protein RepB |
| Mchl_5617 | p1METDI0010 | 8 | *repC* | replication protein C |
| Mchl_5620 | p1METDI0011 | 8 | *-* | conserved protein of unknown function (fragment) |
| Mchl_5622 | p1METDI0012 | 8 | *-* | conserved protein of unknown function |
| Mchl_5644 | p1METDI0053 | 8 | *-* | conserved protein of unknown function, putative PilT domain protein |
| Mchl_5646 | p1METDI0052 | 8 | *-* | putative phage integrase |

**^a^** MaGe annotation (https://www.genoscope.cns.fr/agc/microscope). In bold, genes located on plasmid pCMU01 (Roselli et al., 2013) and p1METDI (Vuilleumier et al., 2009).

**^b^** Conserved synteny of at least 4 co-localized genes on the genomes of strains CM4 and DM4. Numbers refer to synteny groups. The synteny group n°8 is plasmid-located in both strains (plasmid pCMU01 in strain CM4 and plasmid p1METDI in strain DM4)

**Table S4. Genes of the core genome with similar transcription profiles shared between *M. extorquens* strains CM4 and DM4**

| **Group and label^a^** | | **Name** | **Product** | **log**_2_**fc^b^** | | **Adjusted p-value^c^** | |
| --- | --- | --- | --- | --- | --- | --- | --- |
| **CM4** | **DM4** |  |  | **CM4** | **DM4** | **CM4** | **DM4** |
| **More abundant on both chlorinated methanes** | | | | | | | |
| Mchl_0651 | METDI0620 | - | conserved protein of unknown function | 2.3 | 3.8 | <0.001 | <0.001 |
| Mchl_1347 | METDI1765 | - | conserved protein of unknown function | 2.1 | 3.3 | <0.001 | 0.004 |
| Mchl_2116 | METDI2461 | - | conserved protein of unknown function | 2.6 | 2.5 | <0.001 | 0.016 |
| Mchl_2560 | METDI3067 | - | putative methyl-accepting chemotaxis sensory transducer | 2.0 | 2.5 | <0.001 | 0.009 |
| Mchl_2793 | METDI3303 | - | protein of unknown function | 2.7 | 2.8 | <0.001 | <0.001 |
| Mchl_3129 | METDI3671 | - | conserved protein of unknown function, DUF1328 | 2.5 | 2.7 | <0.001 | <0.001 |
| Mchl_3408 | METDI3863 | *hppA* | H+ translocating pyrophosphate synthase | 2.4 | 3.5 | <0.001 | 0.019 |
| Mchl_3885 | METDI4584 | - | protein of unknown function | 2.2 | 2.5 | <0.001 | 0.006 |
| Mchl_3919 | METDI4618 | - | conserved protein of unknown function | 2.4 | 2.9 | <0.001 | <0.001 |
| Mchl_4560 | METDI5190 | - | protein of unknown function | 2.1 | 3.8 | <0.001 | <0.001 |
| Mchl_5157 | METDI5746 | *degP* | periplasmic serine protease (DegP) | 4.6 | 6.6 | <0.001 | <0.001 |
| Mchl_5526 | METDI1593 | - | transposase of ISMex11, IS3 family | 2.3 | 2.1 | <0.001 | 0.010 |
| **More abundant on chloromethane** | | | | | | | |
| Mchl_0812 | METDI0990 | *gcvP* | glycine cleavage complex protein P, PLP-dependent glycine dehydrogenase | 4.6 | 0.4 | < 0.001 | 0.9 ^d^ |
| Mchl_1416 | METDI1841 | - | putative membrane protein | 2.2 | 1.4 | <0.001 | 0.071 |
| Mchl_1525 | METDI1969 | - | conserved protein of unknown function | 2.0 | 1.9 | <0.001 | 0.006 |
| Mchl_2287 | METDI2777 | - | conserved protein of unknown function | 2.2 | 1.9 | <0.001 | 0.083 |
| Mchl_2987 | METDI3526 | *pntAB* | NAD(P)^+^ transhydrogenase, subunit alpha part 2 | 3.6 | 0.1 | 0.001 | 1.000^d^ |
| Mchl_3132 | METDI3674 | - | protein of unknown function | 2.2 | 1.9 | <0.001 | 0.022 |
| Mchl_4484 | METDI5109 | - | protein of unknown function | 4.2 | 1.7 | <0.001 | 0.060 |
| Mchl_5057 | METDI5648 | - | putative seryl-tRNA synthetase | 2.2 | 1.6 | <0.001 | 0.072 |
| Mchl_5058 | METDI5649 | - | acyl-CoA dehydrogenase | 2.2 | 1.7 | <0.001 | 0.036 |
| Mchl_5066 | METDI5657 | - | putative homoserine O-succinyltransferase (MetA) | 2.0 | 1.8 | <0.001 | 0.051 |
| MCv2_5149 | METDI2503 | - | coenzyme PQQ biosynthesis protein A | 2.1 | 1.5 | <0.001 | 0.063 |
| **More abundant on dichloromethane** | | | | | | | |
| Mchl_0009 | METDI0008 | - | protein of unknown function | 1.9 | 2.4 | <0.001 | <0.001 |
| Mchl_0154 | METDI0091 | *iorA* | isoquinoline 1-oxidoreductase, alpha subunit | 0.9 | 2.5 | 0.014 | <0.001 |
| Mchl_0155 | METDI0092 | - | oxidoreductase, molybdenum cofactor binding subunit | 1.5 | 2.9 | <0.001 | <0.001 |
| Mchl_0156 | METDI0093 | - | putative voltage-dependent anion channel | 1.9 | 2.7 | <0.001 | 0.001 |
| Mchl_0171 | METDI0108 | - | protein of unknown function | 1.0 | 2.0 | 0.017 | 0.012 |
| Mchl_0189 | METDI0128 | - | inositol-1-phosphate synthase | 1.7 | 2.0 | <0.001 | 0.003 |
| Mchl_0190 | METDI0129 | - | putative dTDP-glucose 4,6-dehydratase | 1.8 | 2.2 | <0.001 | 0.002 |
| Mchl_0194 | METDI0133 | - | putative glycosyl transferase | 1.3 | 2.6 | 0.048 | 0.006 |
| Mchl_0360 | METDI0190 | - | putative glutathione peroxidase | 1.1 | 2.5 | 0.002 | 0.002 |
| Mchl_1112 | METDI1593 | - | transposase of IS*Mex11*, IS3 family | 0.7 | 2.1 | 0.410 | 0.010 |
| Mchl_1492 | METDI1934 | - | protein of unknown function | 1.7 | 2.2 | <0.001 | 0.014 |
| Mchl_1516 | METDI1959 | - | conserved protein of unknown function precursor | 0.7 | 2.5 | 0.047 | 0.036 |
| Mchl_1729 | METDI2115 | - | conserved protein of unknown function | 0.1 | 3.0 | 0.920 | <0.001 |
| Mchl_2091 | METDI2432 | - | putative aminoglycoside phosphotransferase | 1.7 | 2.7 | <0.001 | <0.001 |
| Mchl_2208 | METDI2693 | - | putative molybdopterin oxidoreductase | 1.9 | 3.8 | <0.001 | 0.008 |
| Mchl_2537 | METDI3043 | - | conserved protein of unknown function with 2 CBS domains | 1.0 | 4.4 | 0.003 | 0.018 |
| Mchl_2848 | METDI3359 | - | protein of unknown function | 1.1 | 2.1 | 0.029 | 0.002 |
| Mchl_2894 | METDI3426 | - | conserved protein of unknown function | 1.3 | 2.1 | <0.001 | 0.006 |
| Mchl_3030 | METDI3569 | *arcB* | ornithine cyclodeaminase | 1.6 | 2.6 | <0.001 | <0.001 |
| Mchl_3696 | METDI4190 | - | protein of unknown function | -1.1 | 2.1 | 0.001 | 0.048 |
| Mchl_3921 | METDI4620 | - | protein of unknown function | 1.2 | 2.2 | 0.027 | 0.002 |
| Mchl_3964 | METDI4670 | - | putative monooxygenase with ATPase activity | 1.8 | 2.0 | <0.001 | 0.003 |
| Mchl_4030 | METDI4699 | - | transcriptional regulator, AraC family | 1.8 | 4.3 | <0.001 | <0.001 |
| Mchl_4284 | METDI4905 | - | putative patatin-like phospholipase | 1.4 | 2.5 | 0.004 | <0.001 |
| Mchl_4335 | METDI4959 | - | conserved protein of unknown function | 1.5 | 2.2 | 0.001 | <0.001 |
| Mchl_4440 | METDI5067 | - | conserved protein of unknown function | -0.7 | 3.0 | 0.092 | 0.002 |
| Mchl_4491 | METDI5117 | - | protein of unknown function | 1.1 | 3.5 | 0.007 | <0.001 |
| Mchl_4519 | METDI5146 | *mxaW* | conserved exported protein of unknown function | 1.0 | 2.1 | 0.029 | 0.001 |
| Mchl_4727 | METDI1224 | - | putative NcrB-like regulator, DUF156 | 0.8 | 2.7 | 0.059 | <0.001 |
| Mchl_4728 | METDI1223 | - | putative NcrA-like major facilitator superfamily permease | 0.9 | 2.1 | 0.034 | 0.009 |
| Mchl_5043 | METDI5633 | *fdh1A* | tungsten-containing formate dehydrogenase alpha subunit | 1.6 | 2.3 | <0.001 | 0.200^d^ |
| Mchl_5285 | METDI5875 | - | putative photosynthesis gene regulator (*bchF*-*crtJ*) | 1.1 | 2.5 | 0.001 | 0.036 |
| Mchl_5301 | METDI5891 | - | putative endonuclease | 1.5 | 3.2 | <0.001 | <0.001 |
| **More abundant on methanol** | | | | | | | |
| Mchl_1165 | METDI1517 | - | conserved protein of unknown function | -3.1 | -2.3 | <0.001 | <0.001 |
| Mchl_1533 | METDI1978 | - | conserved protein of unknown function | -2.9 | -2.1 | <0.001 | 0.037 |
| Mchl_1534 | METDI1979 | - | putative NosX precursor (nitrous oxide reductase) | -3.3 | -3.2 | <0.001 | 0.013 |
| Mchl_1535 | METDI1980 | - | putative regulatory protein NosR | -3.4 | -2.6 | <0.001 | 0.002 |
| Mchl_1536 | METDI1981 | - | conserved protein of unknown function | -3.7 | -2.5 | <0.001 | <0.001 |
| Mchl_1537 | METDI1982 | - | conserved exported protein of unknown function | -3.3 | -3.3 | <0.001 | <0.001 |
| Mchl_1538 | METDI1983 | - | cytochrome c550 protein, putative PQQ-dependent methanol/ethanol oxidation system | -3.9 | -3.8 | <0.001 | <0.001 |
| Mchl_1539 | METDI1984 | - | conserved protein of unknown function, periplasmic binding protein precursor, putative periplasmic binding protein-like II | -4.5 | -3.7 | <0.001 | <0.001 |
| Mchl_3283 | METDI3835 | *dctA* | C4-dicarboxylate transport protein | -2.9 | -3.2 | <0.001 | 0.029 |
| **More abundant on methanol in strain CM4 only** | | | | | | | |
| Mchl_0844 | METDI1024 | - | conserved membrane protein of unknown function | -2.0 | -1.8 | <0.001 | 0.031 |
| Mchl_1540 | METDI1985 | *exaF* | PQQ-dependent methanol/ethanol dehydrogenase | -3.4 | -1,75 | < 0.001 | 0.3 ^d^ |
| **More abundant on methanol in strain DM4 only** | | | | | | | |
| Mchl_0500 | METDI0537 | - | ABC transporter, membrane protein | 1.6 | -2.5 | <0.001 | 0.060 |
| Mchl_0501 | METDI0538 | - | ABC transporter, permease | 1.1 | -2.0 | 0.015 | 0.073 |
| Mchl_1243 | METDI1697 | - | putative formate/nitrate transporter | -1.6 | -3.4 | <0.001 | <0.001 |
| Mchl_1337 | METDI1753 | *eshA* | Nucleotide-binding protein EshA | -1.1 | -3.3 | <0.001 | <0.001 |
| Mchl_1338 | METDI1754 | - | putative cysteine desulfurase (SufS domain) | -1.1 | -2.2 | <0.001 | 0.092 |
| Mchl_1341 | METDI1761 | - | conserved exported protein of unknown function | -1.4 | -2.6 | <0.001 | <0.001 |
| Mchl_1417 | METDI1842 | - | conserved protein of unknown function | -1.0 | -2.1 | 0.015 | 0.005 |
| Mchl_1556 | METDI2005 | - | putative cytochrome c, class I | 0.9 | -3.2 | 0.008 | 0.043 |
| Mchl_1558 | METDI2007 | - | ABC transporter, fused ATPase and permease domains (CydD-like) | 1.8 | -3.4 | <0.001 | <0.001 |
| Mchl_1561 | METDI2011 | *cydB* | cytochrome d terminal oxidase, polypeptide subunit II | 1.5 | -3.4 | <0.001 | 0.036 |
| Mchl_1562 | METDI2012 | - | putative exported protein, putative cyd operon protein | 1.8 | -3.7 | <0.001 | <0.001 |
| Mchl_1717 | METDI2103 | - | conserved protein of unknown function | 2.5 | -2.4 | <0.001 | 0.002 |
| Mchl_2374 | METDI2867 | *glnK* | ABC transporter, fused ATPase and transmembrane permease domains | -0.8 | -2.5 | 0.048 | 0.029 |
| Mchl_2380 | METDI2873 | *fdh4B* | formate dehydrogenase subunit B | -1.2 | -3.2 | 0.001 | <0.001 |
| Mchl_2381 | METDI2874 | *fdh4A* | formate dehydrogenase subunit A | -0.7 | -4.0 | 0.048 | <0.001 |
| Mchl_2653 | METDI3157 | *glnII* | glutamine synthetase II | -0.8 | -3.2 | 0.082 | 0.085 |
| Mchl_3806 | METDI4303 | - | putative transcriptional regulator | 1.5 | -3.6 | <0.001 | 0.048 |
| Mchl_3807 | METDI4304 | - | conserved protein with ANAH-like domain | 1.1 | -3.1 | 0.004 | 0.054 |
| Mchl_3808 | METDI4305 | - | conserved protein of unknown function | 0.8 | -2.4 | 0.069 | 0.038 |
| Mchl_3810 | METDI4307 | - | protein of unknown function | 0.9 | -2.7 | 0.029 | 0.034 |
| Mchl_4141 | METDI4814 | - | protein of unknown function; putative exported protein | 0.9 | -4.0 | 0.096 | 0.001 |
| Mchl_4142 | METDI4816 | *qxtB* | cytochrome bd-quinol oxidase subunit II | 1.2 | -2.7 | 0.008 | 0.060 |
| Mchl_4650 | METDI5284 | - | protein of unknown function | -1.6 | -3.1 | <0.001 | <0.001 |
| Mchl_4958 | METDI5532 | *nrtB* | nitrate transport permease protein | -1.1 | -2.4 | 0.028 | 0.001 |
| Mchl_4959 | METDI5533 | *nrtA* | nitrate transporter component | -1.1 | -2.7 | 0.007 | <0.001 |
| **Not differentially expressed** | | | | | | | |
| Mchl_0016 | METDI0015 | - | protein of unknown function | 1.0 | 1.4 | 0.036 | 0.092 |
| Mchl_0167 | METDI0104 | *coxA* | cytochrome c oxidase subunit I | 0.8 | 1.9 | 0.048 | 0.033 |
| Mchl_0186 | METDI0125 | - | conserved protein of unknown function | 1.6 | 1.8 | <0.001 | 0.029 |
| Mchl_0188 | METDI0127 | - | conserved protein of unknown function | 1.4 | 1.9 | 0.005 | 0.029 |
| Mchl_0192 | METDI0131 | - | conserved protein of unknown function | 1.3 | 1.8 | 0.006 | 0.038 |
| Mchl_0193 | METDI0132 | - | conserved protein of unknown function; putative UDP-glycosyltransferase/ glycogen phosphorylase domain | 1.2 | 1.8 | 0.021 | 0.058 |
| Mchl_0197 | METDI0136 | - | putative UDP-glucose 4-epimerase | 0.8 | 1.6 | 0.050 | 0.054 |
| Mchl_0490 | METDI0526 | - | protein of unknown function | 1.5 | 1.8 | 0.008 | 0.026 |
| Mchl_0729 | METDI0699 | - | protein of unknown function, putative chaperone-like protein | 1.0 | 1.6 | 0.003 | 0.085 |
| Mchl_1015 | METDI1362 | - | protein of unknown function | 1.2 | 1.8 | 0.013 | 0.044 |
| Mchl_1021 | METDI1352 | - | protein of unknown function | 0.8 | 1.9 | 0.072 | 0.027 |
| Mchl_1029 | METDI1362 | - | protein of unknown function | 1.1 | 1.8 | 0.006 | 0.044 |
| Mchl_1052 | METDI1383 | - | putative peptidase S8 and S53 | 1.7 | 1.8 | <0.001 | 0.009 |
| Mchl_1373 | METDI1794 | - | protein of unknown function | 1.1 | 1.9 | 0.010 | 0.043 |
| Mchl_1392 | METDI1815 | - | protein of unknown function | 1.8 | 1.5 | <0.001 | 0.093 |
| Mchl_1526 | METDI1970 | - | putative short-chain dehydrogenase/reductase SDR | 1.2 | 1.5 | 0.004 | 0.059 |
| Mchl_1665 | METDI2048 | - | protein of unknown function | 0.9 | 1.9 | 0.015 | 0.009 |
| Mchl_1713 | METDI2099 | - | precorrin-3B synthase | 1.1 | -1.7 | 0.005 | 0.051 |
| Mchl_1715 | METDI2101 | - | conserved protein of unknown function | 1.2 | -1.9 | 0.001 | 0.026 |
| Mchl_1921 | METDI2307 | *pck* | phosphoenolpyruvate carboxykinase | 1.3 | 1.8 | 0.001 | 0.020 |
| Mchl_2097 | METDI2438 | *msrB* | methionine sulfoxide reductase | 1.1 | 1.6 | 0.035 | 0.036 |
| Mchl_2252 | METDI2739 | - | conserved protein of unknown function | -1.5 | -1.5 | 0.001 | 0.070 |
| Mchl_2256 | METDI2744 | - | protein of unknown function | 1.3 | 1.6 | 0.003 | 0.029 |
| Mchl_2279 | METDI2768 | - | putative L,D-transpeptidase catalytic domain (YkuD) | 1.3 | 1.7 | 0.001 | 0.044 |
| Mchl_2394 | METDI2887 | - | putative two-component system regulator | 1.0 | 1.9 | 0.009 | 0.018 |
| Mchl_2530 | METDI3034 | - | protein of unknown function | 1.3 | 1.9 | 0.008 | 0.006 |
| Mchl_2640 | METDI3144 | - | putative ligase (YgfA) | 1.3 | 1.5 | 0.060 | 0.001 |
| Mchl_2690 | METDI3196 | - | protein of unknown function | 1.9 | 1.4 | <0.001 | 0.075 |
| Mchl_2850 | METDI3360 | - | protein of unknown function | 1.2 | 1.9 | 0.005 | 0.009 |
| Mchl_2978 | METDI3517 | - | conserved protein of unknown function | 1.1 | 1.8 | 0.003 | 0.044 |
| Mchl_3057 | METDI3599 | - | conserved protein of unknown function | -0.8 | -1.6 | 0.098 | 0.056 |
| Mchl_3170 | METDI3713 | - | putative phage-related transcriptional regulator | 0.9 | 1.6 | 0.071 | 0.043 |
| Mchl_3222 | METDI3768 | - | conserved protein of unknown function | -0.7 | -1.7 | 0.064 | 0.071 |
| Mchl_3441 | METDI3904 | - | protein of unknown function | 0.8 | 1.3 | 0.093 | 0.075 |
| Mchl_3615 | METDI4083 | - | protein of unknown function | 1.0 | 1.6 | 0.010 | 0.074 |
| Mchl_3891 | METDI4590 | - | sodium/sulphate symporter | -0.9 | -1.7 | 0.020 | 0.094 |
| Mchl_4149 | METDI4824 | - | protein of unknown function | 1.9 | 1.9 | <0.001 | 0.062 |
| Mchl_4164 | METDI4839 | *ragD* | RND efflux transporter, MFP subunit | 0.9 | 1.8 | 0.069 | 0.011 |
| Mchl_4316 | METDI4940 | - | protein of unknown function | 0.8 | 1.5 | 0.100 | 0.054 |
| Mchl_4379 | METDI5004 | - | conserved protein of unknown function; putative prephenate/arogenate dehydrogenase domain | -1.0 | -1.8 | 0.029 | 0.037 |
| Mchl_4435 | METDI5062 | - | putative methyl-accepting chemotaxis sensory transducer | 0.9 | 1.8 | 0.010 | 0.091 |
| Mchl_4710 | METDI5345 | *cliA* | RND efflux transporter, MFP subunit | 0.1 | -0.2 | <0.001 | 1.000^d^ |
| Mchl_4962 | METDI5536 | - | protein of unknown function | 1.7 | 1.6 | <0.001 | 0.047 |
| Mchl_5052 | METDI5643 | - | conserved protein of unknown function | 1.9 | 1.4 | <0.001 | 0.092 |
| Mchl_5055 | METDI5646 | - | conserved protein of unknown function | 1.5 | 1.8 | <0.001 | 0.036 |
| Mchl_5065 | METDI5656 | *metY* | trans-sulfuration enzyme (O-acetylhomoserine aminocarboxypropyltransferase) | 1.7 | 1.7 | <0.001 | 0.039 |
| Mchl_5086 | METDI5673 | - | putative glycosyl transferase, WecB/TagA/CpsF family | 1.3 | 1.6 | <0.001 | 0.059 |
| Mchl_5087 | METDI5674 | - | putative glycosyl transferase | 1.1 | 1.5 | 0.004 | 0.055 |
| Mchl_5175 | METDI5762 | - | conserved protein of unknown function | 1.9 | 1.8 | <0.001 | 0.015 |
| Mchl_5176 | METDI5763 | - | protein of unknown function | 1.3 | 1.8 | <0.001 | 0.029 |
| MCv2_2498 | METDI2545 | - | putative exported protein | 0.8 | 1.8 | 0.041 | 0.036 |

^a^ MaGe annotation (<https://www.genoscope.cns.fr/agc/microscope>)

^b^ log_2_fc of normalized read numbers between cultures grown with chlorinated methanes and with methanol

^c^ Only genes with adjusted *p*-value < 0.1 (false discovery rate) were considered in the analysis

^d^ Fold change confirmed by RT-qPCR (Fig S2)

**Table S5. Differential transcript abundance in *M. extorquens* CM4 cultures grown with chloromethane or with methanol**

| **Label^a^** | | | **Name** | **Product** | **Occurrence ^b^** | **RNAseq data** | | |
| --- | --- | --- | --- | --- | --- | --- | --- | --- |
|  |  |  |  |  |  | **log_2_fc^c^** | | **Adjusted p-value^d^** |
| **Chlorinated methane utilization** | | | | | | |  | |
|  | | Mchl_5694 | *hutI* | putative imidazolonepropionase | pCMU01 | 7.7 | | < 0.001 |
|  | | Mchl_5696 | *-* | putative regulatory protein FmdB | pCMU01 | 7.7 | | < 0.001 |
|  | | Mchl_5697 | *cmuA* | methyltransferase/corrinoid binding protein CmuA | pCMU01^f^ | 8.3 | | < 0.001 |
|  | | Mchl_5698 | *cmuC2* | putative methyltransferase, CmuC-like protein | pCMU01 | 8.4 | | < 0.001 |
|  | | Mchl_5699 | *purU* | formyltetrahydrofolate hydrolase | pCMU01^f^ | 10.0 | | < 0.001 |
|  | | Mchl_5700 | *folD* | bifunctional methylenetetrahydrofolate dehydrogenase/ methenyltetrahydrofolate cyclohydrolase | pCMU01 | 8.6 | | < 0.001 |
|  | | Mchl_5717 | *paaE-like* | putative oxidoreductase FAD/NAD(P)-binding domain protein | pCMU01^e^ | 8.1 | | < 0.001 |
|  | | Mchl_5726 | *metF2* | methylenetetrahydrofolate reductase | pCMU01 | 9.6 | | < 0.001 |
|  | | Mchl_5727 | *cmuB* | methylcobalamin:H4folate methyltransferase CmuB | pCMU01^e^ | 9.5 | | < 0.001 |
|  | | Mchl_5728 | *cmuC* | putative methyltransferase CmuC | pCMU01 | 9.1 | | < 0.001 |
| **Cofactor of dehalogenase-associated metabolism** | | | | | | |  | |
|  | | Mchl_1718 | *-* | putative cobalt transporter, subunit CbtA | core | 2.5 | | < 0.001 |
|  | | Mchl_1719 | *-* | putative cobalt transporter subunit CbtB | core | 2.7 | | < 0.001 |
|  | | Mchl_2855 | *-* | putative TonB-dependent siderophore receptor | core | 3.4 | | < 0.001 |
|  | | Mchl_5676 | *-* | putative cobalamin outer membrane transporter BtuB | pCMU01^e^ | 6.7 | | < 0.001 |
|  | | Mchl_5677 | *-* | ABC transporter periplasmic binding component, putative vitamin B_12_ transporter subunit BtuF | pCMU01^e^ | 6.0 | | < 0.001 |
|  | | Mchl_5678 | *-* | ABC transporter membrane component, putative vitamin B_12_ transporter subunit BtuC | pCMU01^e^ | 5.3 | | < 0.001 |
|  | | Mchl_5679 | *-* | ABC transporter ATP-binding component, putative vitamin B_12_ transport subunit BtuD | pCMU01^e^ | 5.0 | | < 0.001 |
|  | | Mchl_5681 | *-* | putative P-loop containing nucleoside triphosphate hydrolase | pCMU01^e^ | 4.8 | | < 0.001 |
|  | | Mchl_5682 | *-* | putative TonB-dependent receptor | pCMU01^e^ | 4.4 | | < 0.001 |
|  | | Mchl_5685 | *cobM2* | precorrin-4 C(11)-methyltransferase | pCMU01^e^ | 7.0 | | < 0.001 |
|  | | Mchl_5686 | *-* | putative cobalamin biosynthesis protein CobE | pCMU01^e^ | 4.8 | | < 0.001 |
|  | | Mchl_5687 | *cobL2* | precorrin-6Y C(5,15)-methyltransferase (decarboxylating) | pCMU01^e^ | 4.7 | | < 0.001 |
|  | | Mchl_5689 | *cobJ2* | precorrin-3B C(17)-methyltransferase | pCMU01^e^ | 5.5 | | < 0.001 |
|  | | Mchl_5690 | *cobI2* | precorrin-2 C(20)-methyltransferase | pCMU01^e^ | 5.7 | | < 0.001 |
|  | | Mchl_5691 | *cobH2* | precorrin-8X methylmutase | pCMU01^e,f^ | 5.9 | | < 0.001 |
|  | | Mchl_5692 | *-* | putative cobalamin biosynthesis protein CobN-like domain | pCMU01^e^ | 7.0 | | < 0.001 |
|  | | Mchl_5693 | *-* | conserved exported protein of unknown function, CoxB-related protein | pCMU01^e^ | 8.9 | | < 0.001 |
|  | | Mchl_5701 | *folC2* | putative folylpolyglutamate synthase and dihydrofolate synthase | pCMU01 | 7.8 | | < 0.001 |
|  | | Mchl_5702 | *cobU2* | nicotinate-nucleotide-dimethylbenzimidazole phosphoribosyltransferase | pCMU01 | 6.8 | | < 0.001 |
|  | | Mchl_5714 | *czcB* | RND efflux transporter, putative membrane fusion protein | pCMU01^e^ | 2.5 | | < 0.001 |
|  | | Mchl_5715 | *czcA2* | RND divalent metal cation efflux transporter membrane component, cobalt-zinc-cadmium resistance protein | pCMU01^e^ | 2.3 | | < 0.001 |
|  | | Mchl_5721 | *cobP2* | bifunctional adenosylcobinamide kinase and adenosylcobinamide-phosphate guanylyltransferase | pCMU01 | 6.7 | | < 0.001 |
|  | | Mchl_5722 | *cobO2* | cob(I)yrinic acid a,c-diamide adenosyltransferase | pCMU01 | 6.5 | | < 0.001 |
|  | | Mchl_5723 | *cobQ2* | cobyric acid synthase | pCMU01 | 6.2 | | < 0.001 |
|  | | Mchl_5724 | *cobD2* | cobalamin biosynthesis protein | pCMU01 | 2.3 | | < 0.001 |
|  | | Mchl_5729 | *cbiD* | cobalamin biosynthesis protein, putative cobalt-precorrin-6A synthase | pCMU01 | 4.3 | | < 0.001 |
|  | | Mchl_5730 | *cobC2* | L-threonine-O-3-phosphate decarboxylase domain | pCMU01 | 4.9 | | < 0.001 |
|  | | Mchl_5731 | *cobA* | uroporphyrinogen-III C-methyltransferase | pCMU01 | 5.6 | | < 0.001 |
|  | | Mchl_5732 | *bluB2* | 5,6-dimethylbenzimidazole synthase, putative cob(II)yrinic acid a,c-diamide reductase | pCMU01 | 5.2 | | < 0.001 |
| **C_1_ metabolism** | | | | | | |  | |
|  | | Mchl_0812 | *gcvP* | glycine cleavage complex protein P, PLP-dependent glycine dehydrogenase | core | 4.6 | | < 0.001 |
|  | | Mchl_0813 | *gcvH* | glycine cleavage complex protein H | core | 4.5 | | < 0.001 |
|  | | Mchl_0814 | *gcvT* | glycine cleavage complex protein T, aminomethyltransferase tetrahydrofolate-dependent | core^f^ | 3.0 | | < 0.001 |
|  | | Mchl_1538 | *-* | c-cytochrome c550 protein associated with PQQ-dependent methanol/ethanol ExaF oxidation system | core^e^ | -3.9 | | < 0.001 |
|  | | Mchl_1540 | *exaF* | PQQ-dependent methanol/ethanol dehydrogenase | core^e^ | -3.4 | | < 0.001 |
|  | | Mchl_5683 | *gck2* | glycerate kinase | pCMU01 | 5.1 | | < 0.001 |
|  | | MCv2_5148 | *pqqA* | coenzyme PQQ biosynthesis protein A | core | 2.2 | | < 0.001 |
|  | | MCv2_5149 | *pqqA* | coenzyme PQQ biosynthesis protein A | core | 2.1 | | < 0.001 |
| **Central metabolism** | | | | | | |  | |
|  | | Mchl_1534 | *-* | putative NosX precursor (nitrous oxide reductase) | core^e^ | - 3.3 | | < 0.001 |
|  | | Mchl_5057 | *-* | putative seryl-tRNA synthetase | variable | 2.2 | | < 0.001 |
|  | | Mchl_5058 | *acd* | acyl-CoA dehydrogenase | variable | 2.2 | | < 0.001 |
|  | | Mchl_5060 | *-* | putative monooxygenase, putative flavin: NADH reductase | variable | 2.5 | | < 0.001 |
|  | | Mchl_5066 | *-* | putative homoserine O-succinyltransferase (MetA) | variable | 2.0 | | < 0.001 |
|  | | Mchl_5154 | *-* | aldo/keto reductase family | core | 2.1 | | < 0.001 |
|  | | Mchl_5521 | *acxC* | acetone carboxylase gamma subunit | pCMU01 | 2.5 | | < 0.001 |
|  | | Mchl_5522 | *acxB* | acetone carboxylase alpha subunit | pCMU01 | 2.1 | | < 0.001 |
|  | | Mchl_5523 | *acxA* | acetone carboxylase beta subunit | pCMU01 | 3.7 | | < 0.001 |
|  | | Mchl_R0001 | *-* | tRNA-Thr | core | 2.1 | | < 0.001 |
|  | | Mchl_R0005 | *-* | tRNA-Glu | core | 2.3 | | < 0.001 |
|  | | Mchl_R0006 | *-* | tRNA-Ser | core | 2.0 | | < 0.001 |
|  | | Mchl_R0057 | *-* | tRNA-Thr | core | 2.3 | | < 0.001 |
|  | | Mchl_R0078 | *-* | tRNA-Asn | core | 2.1 | | < 0.001 |
| **Energy and redox balance** | | | | | | | | |
|  | | Mchl_2986 | *pntAA* | NAD(P)^+^ transhydrogenase, subunit alpha part 1 | core^e^ | 2.7 | | < 0.001 |
|  | | Mchl_2987 | *pntAB* | NAD(P)^+^ transhydrogenase, subunit alpha part 2 | core | 3.6 | | < 0.001 |
|  | | Mchl_2988 | *pntB* | NAD(P)^+^ transhydrogenase, subunit beta | core | 3.4 | | < 0.001 |
|  | | Mchl_3408 | *hppA* | H(^+^) translocating pyrophosphate synthase | core | 2.4 | | < 0.001 |
| **Stress response and transport** | | | | | | |  | |
|  | | Mchl_2781 | *-* | putative L,D-transpeptidase catalytic domain (YkuD) | core | 2.5 | | < 0.001 |
|  | | Mchl_3002 | *-* | putative manganese catalase | core**^f^** | 2.1 | | < 0.001 |
|  | | Mchl_3283 | *dctA* | C4-dicarboxylate transport protein | core | -2.9 | | < 0.001 |
|  | | Mchl_4687 | *ada* | fused DNA-binding transcriptional dual regulator, O6-methylguanine-DNA methyltransferase | core | 5.6 | | < 0.001 |
|  | | Mchl_5016 | *ibpA* | small heat shock protein | core | 2.8 | | < 0.001 |
|  | | Mchl_5157 | *degP* | periplasmic serine protease (DegP) | core | 4.6 | | < 0.001 |
|  | | Mchl_5405 | *clcA2* | putative H(^+^)/Cl(^-^) exchange transporter | pCMU01 | 5.5 | | < 0.001 |
|  | | Mchl_5500 | *-* | transporter, major facilitator superfamily | pCMU01^e^ | 4.4 | | < 0.001 |
| **Regulation** | | | | | | | | |
|  | Mchl_0472 | | - | putative PYP-like sensor domain (PAS domain) | core | 4.8 | | < 0.001 |
|  | Mchl_1288 | | - | putative transcriptional regulator, AraC family | variable | 2.2 | | < 0.001 |
|  | Mchl_2560 | | *-* | putative exported sensory transducer protein | core | 2.0 | | < 0.001 |
|  | Mchl_2920 | | *-* | putative exported sensory transducer protein | variable | -2.4 | | < 0.001 |
|  | Mchl_5499 | | *-* | response regulator in two-component regulatory system | pCMU01 | 2.4 | | < 0.001 |
|  | Mchl_5720 | | *-* | putative methyl-accepting signaling domain protein | pCMU01 | 7.2 | | < 0.001 |
|  | | Rmisc_RNA_1 | *-* | cobalamin riboswitch upstream of *btuB* (Mchl_5676) | pCMU01 | 5.9 | | < 0.001 |
|  | | Rmisc_RNA_4 | *-* | cobalamin riboswitch upstream of gene encoding Mchl_1720 | core | 3.1 | | < 0.001 |
|  | | Rmisc_RNA_14 | *-* | glycine riboswitch upstream of *gcvT* | core | 2.2 | | < 0.001 |
|  | | Rmisc_RNA_15 | *-* | glycine riboswitch upstream of *gcvT* | core | 2.2 | | < 0.001 |
| **Mobile elements and protein of unknown function** | | | | | | |  | |
|  | MCv2_0131 | | - | protein of unknown function, Mchl_0106 upstream gene | specific | 2.3 | | < 0.001 |
|  | Mchl_0106 | | - | conserved protein of unknown function | specific | 2.2 | | < 0.001 |
|  | Mchl_0127 | | - | conserved protein of unknown function | variable | 2.1 | | < 0.001 |
|  | Mchl_0431 | | - | putative exported protein of unknown function | variable | 7.1 | | < 0.001 |
|  | Mchl_0473 | | - | conserved protein of unknown function | core | 3.5 | | < 0.001 |
|  | Mchl_0651 | | - | conserved exported protein of unknown function | core | 2.3 | | < 0.001 |
|  | Mchl_0844 | | - | conserved membrane protein of unknown function | core | -2.0 | | < 0.001 |
|  | Mchl_1165 | | - | conserved membrane protein of unknown function | core | -3.1 | | < 0.001 |
|  | Mchl_1258 | | - | conserved protein of unknown function | variable | 2.9 | | 0.003 |
|  | Mchl_1287 | | - | conserved exported protein of unknown function | specific | 3.0 | | < 0.001 |
|  | Mchl_1347 | | - | conserved protein of unknown function | core | 2.1 | | < 0.001 |
|  | Mchl_1353 | | - | conserved protein of unknown function | variable | 2.9 | | < 0.001 |
|  | Mchl_1416 | | - | putative membrane protein | core | 2.2 | | < 0.001 |
|  | Mchl_1525 | | - | protein of unknown function | variable | 2.0 | | < 0.001 |
|  | Mchl_1533 | | *-* | protein of unknown function | core^e^ | -2.9 | | < 0.001 |
|  | Mchl_1535 | | *-* | conserved membrane protein, FMN-binding & 4Fe-4S binding domains | core^e^ | -3.4 | | < 0.001 |
|  | Mchl_1536 | | *-* | conserved protein of unknown function, putative YVTN beta-propeller repeat family protein | core^e^ | -3.7 | | < 0.001 |
|  | Mchl_1537 | | *-* | conserved exported protein of unknown function | core^e^ | -3.3 | | < 0.001 |
|  | Mchl_1539 | | *-* | conserved protein of unknown function, putative periplasmic binding protein-like II | core^e^ | -4.5 | | < 0.001 |
|  | Mchl_1675 | | - | conserved protein of unknown function | core | 3.4 | | < 0.001 |
|  | Mchl_1687 | | - | protein of unknown function | variable | 2.3 | | 0.032 |
|  | Mchl_1688 | | - | protein of unknown function | core | 2.0 | | 0.002 |
|  | Mchl_1717 | | - | conserved protein of unknown function | core | 2.5 | | < 0.001 |
|  | Mchl_2115 | | - | putative exported protein of unknown function | variable | 2.5 | | < 0.001 |
|  | Mchl_2116 | | - | conserved protein of unknown function | variable | 2.6 | | < 0.001 |
|  | Mchl_R0048 | | - | protein of unknown function | specific | 2.1 | | < 0.001 |
|  | Mchl_2287 | | - | conserved exported protein of unknown function | core | 2.2 | | < 0.001 |
|  | MCv2_2727 | | - | protein of unknown function | specific | -2,3 | | < 0.001 |
|  | Mchl_2793 | | - | protein of unknown function | core | 2.7 | | < 0.001 |
|  | Mchl_2854 | | - | putative exported protein of unknown function | variable | 3.3 | | < 0.001 |
|  | MCv2_3497 | | - | protein of unknown function, *phyR* adjacent gene | specific | 2.6 | | < 0.001 |
|  | Mchl_3129 | | *-* | conserved protein of unknown function, DUF1328 | core | 2.5 | | < 0.001 |
|  | Mchl_3132 | | - | putative exported protein of unknown function | core | 2.2 | | < 0.001 |
|  | Mchl_3370 | | - | protein of unknown function | specific | 2.1 | | < 0.001 |
|  | MCv2_4066 | | - | protein of unknown function, Mchl_3634 upstream gene | specific | 2.1 | | < 0.001 |
|  | Mchl_3634 | | - | putative exported protein of unknown function | core | 2.2 | | < 0.001 |
|  | Mchl_3827 | | - | conserved protein of unknown function | variable | 2.1 | | < 0.001 |
|  | Mchl_3885 | | - | protein of unknown function | core | 2.2 | | < 0.001 |
|  | Mchl_3919 | | - | conserved protein of unknown function | core | 2.4 | | < 0.001 |
|  | Mchl_4484 | | - | putative exported protein of unknown function | core | 4.2 | | < 0.001 |
|  | Mchl_4560 | | - | protein of unknown function | variable | 2.1 | | < 0.001 |
|  | Mchl_4776 | | *-* | conserved protein of unknown function, putative esterase | specific (GEI107) | 2.1 | | < 0.001 |
|  | Mchl_4777 | | *-* | putative outer-membrane porin | variable (GEI107) | 2.1 | | < 0.001 |
|  | Mchl_4779 | | *-* | conserved protein of unknown function | specific (GEI107) | 2.4 | | < 0.001 |
|  | Mchl_4854 | | - | conserved protein of unknown function | specific | 2.2 | | 0.011 |
|  | Mchl_4985 | | - | protein of unknown function precursor | core | 2.1 | | < 0.001 |
|  | Mchl_5172 | | - | conserved protein of unknown function | variable | 2.0 | | < 0.001 |
|  | Mchl_5174 | | - | protein of unknown function | variable | 2.0 | | < 0.001 |
|  | Mchl_5347 | | *-* | conserved protein of unknown function, DUF892 | core | 2.1 | | < 0.001 |
|  | Mchl_5465 | | *-* | transposase (fragment) | pCMU01 | 2.3 | | < 0.001 |
|  | Mchl_5505 | | *-* | integrase catalytic region | pCMU01 | 2.9 | | 0.018 |
|  | Mchl_5526 | | *-* | transposase of IS*Mex11*, IS*3* family (ORF 2) | pCMU01 | 2.3 | | < 0.001 |
|  | Mchl_5581 | | *-* | transposase (fragment) | pCMU01 | 2.3 | | 0.011 |
|  | Mchl_5618 | | *-* | conserved protein of unknown function (fragment) | pCMU01 | 2.2 | | < 0.001 |
|  | Mchl_5663 | | *-* | conserved protein of unknown function | pCMU01 | 2.1 | | < 0.001 |
|  | Mchl_5680 | | *-* | protein of unknown function | pCMU01 | 4.8 | | < 0.001 |
|  | Mchl_5684 | | *-* | putative transposase (fragment) | pCMU01 | 4.2 | | < 0.001 |
|  | Mchl_5703 | | *-* | putative transposase (fragment) | pCMU01 | 2.4 | | 0.015 |
|  | Mchl_5704 | | *-* | putative transposon (fragment) | pCMU01 | 3.2 | | < 0.001 |
|  | Mchl_5716 | | - | conserved protein of unknown function, CobS-like domain | pCMU01 | 7.1 | | < 0.001 |
|  | Mchl_5718 | | *-* | putative transport protein | pCMU01 | 7.1 | | < 0.001 |
|  | Mchl_5719 | | *-* | putative transport protein | pCMU01 | 7.7 | | < 0.001 |
|  | Mchl_5743 | | - | conserved protein of unknown function | pCMU01 | 2.6 | | 0.002 |
|  | p1MCv2_0094 | | - | protein of unknown function | pCMU01 | 2.3 | | < 0.001 |
|  | p1MCv2_0095 | | - | conserved protein of unknown function | pCMU01 | 2.3 | | < 0.001 |
|  | p1MCv2_0379 | | - | protein of unknown function | pCMU01 | 2.4 | | < 0.001 |
|  | p1MCv2_0380 | | - | protein of unknown function | pCMU01 | 2.1 | | < 0.001 |

^a^ MaGe annotation (<https://www.genoscope.cns.fr/agc/microscope>).

^b^ Genes labeled as core are found in all *M. extorquens* genomes of strains AM1, BJ001, CM4, DM4 and PA1 (80% of identity on at least 80% of the protein length). Genes labeled as pCMU01 are located on the 380 kb plasmid and some have chromosomal gene copies described previously (Roselli et al., 2013). Genes labeled as GEI are located on genomic islands defined in Table S4.

^c^ log_2_ fold-change of normalized read numbers in cultures grown with chloromethane compared to with methanol.

^d^ False discovery rate, only adjusted p-value under 0.1 were considered.

^e^ Part of gene clusters: Mchl_1533-1540; Mchl_5676-5693 gene; Mchl_5714-5716; Mchl_5499-5500.

^f^ Corresponding encoded protein found more abundant in cell protein extracts of CM4 strain grown with chloromethane versus methanol (Roselli et al., 2013).

**Table S6.** Differential transcript abundance in *M. extorquens* DM4 cultures grown with dichloromethane or with methanol

| **Label^a^** | | | | **Name** | **Product** | | **Occurrence^b^** | **RNAseq data** | | |
| --- | --- | --- | --- | --- | --- | --- | --- | --- | --- | --- |
|  |  |  |  |  |  |  |  | **log_2_fc^c^** | **Adjusted p-value^d^** | |
| **Chlorinated methane utilization** | | | | | |  | | | | |
|  | METDI2655 | | | *dcmR* | transcriptional repressor of DCM dehalogenase | | *dcm* GEI^g^ | 2.7 | 0.040 | |
|  | METDI2656 | | | *dcmA* | dichloromethane dehalogenase (DCM dehalogenase) | | *dcm* GEI^g^ | 4.3 | < 0.001 | |
|  | METDI2657 | | | *-* | conserved protein of unknown function DcmB | | *dcm* GEI | 4.0 | < 0.001 | |
|  | METDI2658 | | | *-* | conserved protein of unknown function DcmC | | *dcm* GEI | 3.5 | < 0.001 | |
| **Cofactor of dehalogenase associated metabolism** | | | | | |  | | | | |
|  | METDI0190 | | | *-* | putative glutathione peroxidase | | core | 2.5 | 0.001 | |
| **C_1_ metabolism** | | | | | | | | | | |
|  | METDI1697 | | | *-* | putative formate/nitrate transporter | | variable | -3.4 | <0.001 | |
|  | METDI2873 | | | *fdh4B* | formate dehydrogenase subunit B | | core | -3.2 | < 0.001 | |
|  | METDI2874 | | | *fdh4A* | formate dehydrogenase subunit A | | core | -4.0 | < 0.001 | |
|  | METDI5146 | | | *mxaW* | conserved protein associated to the methanol dehydrogenase | | core | 2.1 | 0.002 | |
| **Central metabolism** | | | | | |  | | | | |
|  | METDI0091 | | | *-* | oxidoreductase, 2Fe-2S subunit | | core^e^ | 2.5 | < 0.001 | |
|  | METDI0092 | | | *-* | oxidoreductase, molybdenum cofactor binding subunit | | core^e^ | 2.9 | < 0.001 | |
|  | METDI0128 | | | *ino* | inositol-3-phosphate synthase | | core | 2.1 | 0.003 | |
|  | METDI0129 | | | *-* | putative dTDP-glucose 4,6-dehydratase | | core | 2.2 | 0.002 | |
|  | METDI0133 | | | *-* | putative glycosyl transferase | | variable | 2.6 | 0.006 | |
|  | METDI0260 | | | *-* | glucokinase (C-terminal fragment) | | variable, GEI160 | -4.3 | < 0.001 | |
|  | METDI0262 | | | *-* | glucokinase (N-terminal fragment) | | variable, GEI160 | -2.6 | 0.016 | |
|  | METDI0283 | | | *adh-like* | putative alcohol dehydrogenase | | variable, GEI160 | -3.3 | 0.029 | |
|  | METDI0286 | | | *xfp* | D-xylulose 5-phosphate/ D-fructose 6-phosphate phosphoketolase | | variable, GEI160 | -2.9 | 0.099 | |
|  | METDI0292 | | | *-* | putative poly-beta-hydroxyalkanoate synthase (PhbC) | | specific, GEI160 | -3.1 | 0.085 | |
|  | METDI0293 | | | *fabI* | enoyl-(acyl-carrier-protein) reductase (NADH) | | specific, GEI160 | -3.6 | 0.046 | |
|  | METDI0294 | | | *-* | putative phosphate butyryltransferase | | specific, GEI160 | -3.6 | 0.045 | |
|  | METDI0295 | | | *-* | putative acetate kinase (AckA) | | specific, GEI160 | -3.4 | <0.001 | |
|  | METDI0333 | | | *-* | putative haloacid dehalogenase-like hydrolase | | specific, GEI160 | -2.7 | < 0.001 | |
|  | METDI0349 | | | *-* | putative protein disulfide reductase/isomerase, putative thioredoxin | | specific, GEI160 | -2.4 | 0.057 | |
|  | METDI1754 | | | *-* | putative cysteine desulfurase (SufS domain) | | variable | -2.2 | 0.094 | |
|  | METDI1770 | | | *ohyA* | oleate hydratase | | variable | -3.4 | 0.044 | |
|  | METDI2432 | | | *-* | putative phosphotransferase | | core | 2.7 | < 0.001 | |
|  | METDI2693 | | | *-* | putative molybdopterin oxidoreductase | | core | 3.8 | 0.008 | |
|  | METDI3157 | | | *glnII* | glutamine synthetase, type II | | core | -3.2 | 0.084 | |
|  | METDI3569 | | | *arcB* | ornithine cyclodeaminase | | core^f^ | 2.6 | < 0.001 | |
|  | METDI4522 | | | *-* | putative glucokinase (C-terminal fragment) | | specific, GEI197 | -3.5 | < 0.001 | |
|  | METDI4524 | | | *-* | putative glucokinase (N-terminal fragment) | | variable, GEI197 | -2.6 | 0.035 | |
|  | METDI4527 | | | *-* | putative biotin/lipoyl attachment domain | | variable, GEI197 | -2.8 | 0.054 | |
|  | METDI4540 | | | *adh-like* | putative alcohol dehydrogenase | | variable, GEI197 | -3.7 | 0.012 | |
|  | METDI4543 | | | *xfp* | D-xylulose 5-phosphate/D-fructose 6-phosphate phosphoketolase | | variable, GEI197 | -3.1 | 0.085 | |
|  | METDI4544 | | | *-* | putative acetate kinase (partial) | | variable, GEI197 | -5.0 | < 0.001 | |
|  | METDI4670 | | | *-* | putative monooxygenase with ATPase activity | | core | 2.0 | 0.003 | |
|  | METDI4905 | | | *-* | putative patatin-like phospholipase | | core | 2.5 | < 0.001 | |
| **Energy and redox balance** | | | | | | | | | | |
|  | METDI0275 | | | *-* | putative cytochrome c | | variable, GEI160 | -2.9 | 0.045 | |
|  | METDI0301 | | | *-* | cation-transporting ATPase (P-type) | | variable, GEI160 | -3.3 | 0.071 | |
|  | METDI0322 | | | *-* | putative cytochrome c, class I | | specific, GEI160 | -2.2 | 0.034 | |
|  | METDI0363 | | | *-* | putative cytochrome c oxidase, subunit I | | specific, GEI160 | -2.6 | 0.011 | |
|  | METDI0364 | | | *-* | putative cytochrome c oxidase, subunit II (CbaB) | | specific, GEI160 | -3.4 | < 0.001 | |
|  | METDI0366 | | | *-* | putative cytochrome c | | specific, GEI160 | -2.9 | 0.001 | |
|  | METDI0367 | | | *-* | putative cytochrome c | | specific, GEI160 | -2.7 | < 0.001 | |
|  | METDI1983 | | | *-* | cytochrome c550 | | core | -3.8 | < 0.001 | |
|  | METDI2005 | | | *-* | putative cytochrome c, class I | | core | -3.2 | 0.042 | |
|  | METDI2007 | | | *-* | ABC transporter, fused ATPase and permease domains (CydD-like) | | core | -3.4 | < 0.001 | |
|  | METDI2009 | | | *-* | ABC transporter related, fused ATPase and permease domains (CydD-like) | | core | -3.3 | < 0.001 | |
|  | METDI2011 | | | *cydB* | cytochrome d terminal oxidase, polypeptide subunit II | | core^e^ | -3.4 | 0.036 | |
|  | METDI2012 | | | *-* | putative exported *cyd* operon protein | | specific | -3.7 | < 0.001 | |
|  | METDI3863 | | | *hppA* | H^+^ translocating pyrophosphate synthase | | core^f^ | 3.5 | 0.020 | |
|  | METDI4503 | | | - | putative cytochrome c oxidase, subunit I | | specific, GEI197 | -2.4 | < 0.001 | |
|  | METDI4504 | | | - | putative cytochrome c oxidase, subunit II | | specific, GEI197 | -3.0 | < 0.001 | |
|  | METDI4506 | | | *-* | putative cytochrome c, putative exported protein | | specific, GEI197 | -2.5 | 0.006 | |
|  | METDI4507 | | | *-* | putative cytochrome c | | specific, GEI197 | -3.3 | < 0.001 | |
|  | METDI4538 | | | *-* | putative cytochrome c, class I | | variable, GEI197 | -2.8 | 0.034 | |
|  | METDI4554 | | | *-* | cation-transporting ATPase (P-type) | | variable, GEI197 | -3.5 | 0.059 | |
|  | METDI4564 | | | *-* | putative cytochrome c, class I | | variable, GEI197 | -2.6 | 0.003 | |
|  | METDI4816 | | | *qxtB* | cytochrome bd-quinol oxidase subunit II | | core^e^ | -2.7 | 0.061 | |
| **Stress response and transport** | | | | | | | | | | |
|  | METDI0093 | | | *-* | putative voltage-dependent anion channel | | core^e^ | 2.7 | 0.001 | |
|  | METDI0255 | | | *-* | RND efflux transporter, MFP subunit | | specific, GEI160 | -2.1 | 0.084 | |
|  | METDI0288 | | | *clpB* | protein disaggregation chaperone | | core, GEI160 | -3.6 | 0.014 | |
|  | METDI0266 | | | *-* | putative ion transport domain | | variable, GEI160 | -3.1 | < 0.001 | |
|  | METDI0289 | | | *-* | putative heat shock protein DnaJ | | specific, GEI160 | -3.9 | 0.008 | |
|  | METDI0269 | | | *-* | putative manganese transporter | | variable, GEI160 | -3.3 | < 0.001 | |
|  | METDI0290 | | | *-* | DnaJ-associated protein of unknown function | | specific, GEI160 | -4.0 | 0.001 | |
|  | METDI0329 | | | *-* | RND efflux transporter, MFP subunit | | specific, GEI160 | -3.0 | 0.062 | |
|  | METDI0330 | | | *-* | RND efflux transporter, HME family, translocase subunit | | variable, GEI160 | -3.0 | < 0.001 | |
|  | METDI0537 | | | *-* | ABC transporter, fused tandem ATPase and permease domains | | core | -2.5 | 0.059 | |
|  | METDI0538 | | | *-* | ABC-2 transporter, permease | | core | -2.0 | 0.073 | |
|  | METDI1223 | | | *-* | putative cobalt/nickel resistance NcrA-like major facilitator superfamily permease | | variable | 2.1 | 0.009 | |
|  | METDI1414 | | | *ardC* | antirestriction protein (ArdC) | | specific | -2.2 | 0.026 | |
|  | METDI3043 | | | *-* | conserved protein of unknown function with 2 CBS domains | | core | 4.4 | 0.002 | |
|  | METDI3835 | | | *dctA* | C4-dicarboxylate transport protein | | core | -3.2 | 0.020 | |
|  | METDI4514 | | | *-* | putative transporter, SulP family | | specific, GEI197 | -3.3 | 0.076 | |
|  | METDI4528 | | | *-* | putative ion transport domain | | variable, GEI197 | -3.1 | < 0.001 | |
|  | METDI4531 | | | *-* | putative manganese transporter | | variable, GEI197 | -3.3 | 0.051 | |
|  | METDI4470 | | | *-* | putative L,D-transpeptidase catalytic domain (YkuD) | | specific, GEI197 | -2.3 | <0.001 | |
|  | METDI4695 | | | *opuAA* | glycine/betaine/proline ABC transporter, ATP-binding component | | variable | 2.3 | 0.001 | |
|  | METDI4696 | | | *opuAB* | glycine/betaine/proline ABC transporter, membrane component | | variable | 2.0 | 0.016 | |
|  | METDI4813 | | | *-* | RND efflux transporter, MFP subunit | | core | -3.6 | < 0.001 | |
|  | METDI5532 | | | *nrtB* | nitrate transport permease protein | | core^e^ | -2.4 | 0.001 | |
|  | METDI5533 | | | *nrtA* | nitrate transporter component | | core^e^ | -2.7 | <0.001 | |
|  | METDI5746 | | | *degP* | periplasmic serine protease (DegP) | | core | 6.6 | 0.010 | |
|  | METDI5891 | | | *-* | putative endonuclease | | core | 3.2 | < 0.001 | |
| **Regulation** | | | | | | | | | | |
|  | | METDI0386 | | *-* | histone deacetylase family protein | | variable, GEI160 | -2.9 | 0.029 | |
|  | | METDI1224 | | *-* | putative cobalt/nickel resistance NcrB-like regulator | | variable | 2.8 | < 0.001 | |
|  | | METDI1753 | | *eshA* | nucleotide-binding protein EshA | | core | -3.3 | < 0.001 | |
|  | | METDI2867 | | *glnK* | nitrogen regulatory protein P-II | | core^e^ | -2.5 | 0.029 | |
|  | | METDI3067 | | *-* | putative methyl-accepting chemotaxis sensory transducer | | core | 2.5 | 0.009 | |
|  | | METDI4303 | | *-* | putative transcriptional regulator, Crp/Fnr family | | variable | -3.6 | 0.048 | |
|  | | METDI4515 | | *-* | histone deacetylase family protein | | variable, GEI197 | -3.8 | 0.033 | |
|  | | METDI4520 | | *-* | putative transcriptional regulator, Crp/Fnr family | | variable, GEI197 | -3.2 | 0.072 | |
|  | | METDI4699 | | *-* | transcriptional regulator, AraC family | | core^e^ | 4.3 | < 0.001 | |
|  | | METDI4741 | | *-* | putative response regulator (CheY-like protein) | | core | 2.0 | 0.009 | |
|  | | METDI5068 | | *-* | putative transcriptional regulator | | variable^f^ | 3.2 | 0.011 | |
|  | | METDI5875 | | *-* | putative photosynthesis gene regulator (*bchF-crtJ*) | | variable | 2.5 | 0.004 | |
|  | | **Mobile elements and protein of unknown function** | | | | | | | | |
|  | | | METDI0008 | *-* | protein of unknown function | | core | 2.4 | < 0.001 |  |
|  | | | METDI0108 | *-* | protein of unknown function | | variable | 2.0 | 0.012 |  |
|  | | | METDI0263 | *-* | protein of unknown function | | specific | -2.7 | 0.003 |  |
|  | | | METDI0267 | *-* | conserved protein of unknown function | | variable | -2.8 | 0.007 |  |
|  | | | METDI0270 | *-* | protein of unknown function | | variable | -2.6 | 0.094 |  |
|  | | | METDI0285 | *-* | protein of unknown function | | specific | -3.3 | 0.048 |  |
|  | | | METDI0300 | *-* | protein of unknown function | | specific | -2.6 | 0.029 |  |
|  | | | METDI0306 | *-* | protein of unknown function | | specific | -2.7 | < 0.001 |  |
|  | | | METDI0334 | *-* | exported protein of unknown function | | specific | -2.2 | 0.017 |  |
|  | | | METDI0344 | *-* | conserved protein of unknown function | | variable | 2.2 | 0.012 |  |
|  | | | METDI0365 | *-* | conserved membane protein of unknown function | | specific, GEI160 | -3.3 | < 0.001 |  |
|  | | | METDI0384 | *-* | protein of unknown function | | specific | -2.5 | 0.004 |  |
|  | | | METDI0620 | *-* | conserved exported protein of unknown function | | core | 3.8 | < 0.001 |  |
|  | | | METDI1416 | *-* | transposase of IS*Mdi10*, IS*110* family | | specific | -3.7 | 0.008 |  |
|  | | | METDI1418 | *-* | exported protein of unknown function | | specific | -3.5 | 0.059 |  |
|  | | | METDI1419 | *-* | protein of unknown function | | specific | -3.6 | 0.046 |  |
|  | | | METDI1420 | *-* | protein of unknown function | | specific | -2.9 | 0.084 |  |
|  | | | METDI1446 | *-* | protein of unknown function | | specific | -2.8 | 0.003 |  |
|  | | | METDI1517 | *-* | conserved membrane protein of unknown function | | core | -2.3 | < 0.001 |  |
|  | | | METDI1593 | *-* | transposase of IS*Mex11*, IS*3* family | | variable | 2.1 | 0.009 |  |
|  | | | METDI1597 | *-* | exported protein of unknown function | | specific | 2.3 | 0.001 |  |
|  | | | METDI1598 | *-* | protein of unknown function | | specific | 2.1 | 0.001 |  |
|  | | | METDI1755 | *-* | conserved protein of unknown function | | specific | 2.1 | 0.004 |  |
|  | | | METDI1761 | *-* | conserved exported protein of unknown function | | variable | -2.6 | < 0.001 |  |
|  | | | METDI1762 | *-* | conserved protein of unknown function, DUF1236 | | variable | -2.1 | 0.007 |  |
|  | | | METDI1765 | *-* | protein of unknown function | | core | 3.3 | 0.004 |  |
|  | | | METDI1769 | *-* | conserved protein of unknown function | | core | -3.7 | 0.034 |  |
|  | | | METDI1842 | *-* | conserved protein of unknown function | | core | -2.1 | 0.005 |  |
|  | | | METDI1934 | *-* | protein of unknown function | | variable | 2.2 | 0.014 |  |
|  | | | METDI1959 | *-* | conserved protein of unknown function precursor | | variable | 2.5 | 0.036 |  |
|  | | | METDI1978 | *-* | conserved protein of unknown function | | core | -2.1 | 0.004 |  |
|  | | | METDI1979 | *-* | conserved protein of unknown function, NosX-related protein | | core | -3.2 | 0.012 |  |
|  | | | METDI1980 | *-* | conserved membrane protein, FMN- and 4Fe-4S binding domains | | core | -2.6 | 0.002 |  |
|  | | | METDI1981 | *-* | conserved exported protein of unknown function | | core | -2.5 | < 0.001 |  |
|  | | | METDI1982 | *-* | conserved exported protein of unknown function | | core | -3.3 | < 0.001 |  |
|  | | | METDI1984 | *-* | conserved periplasmic protein (gene adjacent to *exaF*) | | core | -3.8 | < 0.001 |  |
|  | | | METDI2004 | *-* | conserved protein with ANAH-like domain | | core | -3.3 | 0.046 |  |
|  | | | METDI2103 | *-* | conserved protein of unknown function | | core | -2.4 | 0.003 |  |
|  | | | METDI2115 | *-* | conserved exported protein of unknown function | | variable | 3.0 | < 0.001 |  |
|  | | | METDI2461 | *-* | protein of unknown function | | specific | 2.5 | 0.016 |  |
|  | | | METDI3303 | *-* | putative extracellular protein with a calcium binding domain | | core | 2.8 | 0.001 |  |
|  | | | METDI3359 | *-* | protein of unknown function | | core | 2.1 | 0.002 |  |
|  | | | METDI3426 | *-* | conserved protein of unknown function | | core | 2.1 | 0.006 |  |
|  | | | METDI3671 | *-* | conserved membrane protein of unknown function | | core | 2.7 | < 0.001 |  |
|  | | | METDI3864 | *-* | protein of unknown function (gene adjacent to *hppA*) | | specific | 2.4 | 0.004 |  |
|  | | | METDI4190 | *-* | exported protein of unknown function | | core | 2.1 | 0.048 |  |
|  | | | METDI4261 | *-* | conserved protein of unknown function | | core | -2.6 | 0.001 |  |
|  | | | METDI4304 | *-* | conserved protein with ANAH-like domain | | core | -3.1 | 0.054 |  |
|  | | | METDI4305 | *-* | conserved protein of unknown function | | variable | -2.4 | 0.008 |  |
|  | | | METDI4307 | *-* | protein of unknown function | | variable | -2.7 | 0.034 |  |
|  | | | METDI4308 | *-* | protein of unknown function | | variable | -3.4 | < 0.001 |  |
|  | | | METDI4310 | *-* | protein of unknown function | | specific | -2.7 | 0.003 |  |
|  | | | METDI4320 |  | putative alcohol dehydrogenase (fragment) | | specific, GEI197 | 2.6 | 0.064 |  |
|  | | | METDI4505 | *-* | conserved membrane protein of unknown function | | specific | -2.5 | 0.034 |  |
|  | | | METDI4517 | *-* | protein of unknown function | | specific | -2.5 | 0.007 |  |
|  | | | METDI4525 | *-* | protein of unknown function | | specific | -2.8 | 0.046 |  |
|  | | | METDI4526 | *-* | conserved protein of unknown function | | variable | -3.0 | 0.075 |  |
|  | | | METDI4530 | *-* | conserved protein of unknown function | | variable | -3.0 | 0.034 |  |
|  | | | METDI4536 | *-* | protein of unknown function | | variable | -2.4 | 0.011 |  |
|  | | | METDI4545 | *-* | protein of unknown function | | variable | -3.6 | 0.044 |  |
|  | | | METDI4547 | *-* | protein of unknown function | | specific | -3.3 | 0.003 |  |
|  | | | METDI4548 | *-* | conserved protein of unknown function | | variable | -3.2 | 0.083 |  |
|  | | | METDI4552 | *-* | protein of unknown function | | variable | -3.5 | 0.036 |  |
|  | | | METDI4553 | *-* | exported protein of unknown function | | variable | -2.9 | 0.003 |  |
|  | | | METDI4555 | *-* | conserved protein with ANAH-like domain | | variable | -3.3 | 0.001 |  |
|  | | | METDI4557 | *-* | exported protein of unknown function | | variable | -3.4 | < 0.001 |  |
|  | | | METDI4563 | *-* | conserved protein with ANAH-like domain | | variable | -3.3 | 0.051 |  |
|  | | | METDI4565 | *-* | conserved protein with ANAH-like domain | | variable | -2.9 | 0.051 |  |
|  | | | METDI4584 | *-* | protein of unknown function | | core | 2.5 | 0.006 |  |
|  | | | METDI4608 | *-* | conserved membrane protein of unknown function | | variable | 2.2 | 0.006 |  |
|  | | | METDI4618 | *-* | conserved protein of unknown function | | core | 2.9 | 0.004 |  |
|  | | | METDI4620 | *-* | protein of unknown function | | variable | 2.2 | 0.002 |  |
|  | | | METDI4814 | *-* | exported protein of unknown function | | variable | -4.0 | 0.001 |  |
|  | | | METDI4815 | *-* | protein of unknown function | | variable | -2.7 | 0.003 |  |
|  | | | METDI4959 | *-* | conserved protein of unknown function | | core | 2.2 | < 0.001 |  |
|  | | | METDI5067 | *-* | conserved protein of unknown function | | core | 3.0 | 0.002 |  |
|  | | | METDI5117 | *-* | protein of unknown function | | variable | 3.5 | < 0.001 |  |
|  | | | METDI5190 | *-* | protein of unknown function | | variable | 3.8 | 0.001 |  |
|  | | | METDI5265 | *-* | protein of unknown function | | variable | 2.4 | 0.002 |  |
|  | | | METDI5284 | *-* | membrane protein of unknown function | | core | -3.1 | < 0.001 |  |
|  | | | p2METDI0004 | *-* | conserved protein of unknown function | | specific | -3.7 | 0.002 |  |
|  | | | p2METDI0005 | *-* | protein of unknown function | | specific | -3.1 | 0.005 |  |

^a^ MaGe annotation (https://www.genoscope.cns.fr/agc/microscope).

^b^ Genes labeled as core, variable or specific are respectively found in all, at least 2 genomes of *M. extorquens* genomes of strains AM1, BJ001, CM4, DM4 and PA1 (80% of identity on at least 80% of the protein length), or only in the genome of strain DM4.

^c^ log_2_ fold-change of normalized read numbers in cultures grown with dichloromethane compared to with methanol.

^d^ False discovery rate, only adjusted p-value under 0.1 were considered.

^e^ Part of gene clusters: METDI0091-0093; METDI1978-1985; METDI2004-2012; METDI2867-2874; METDI4695-4699; METDI4813-4816; METDI5532-5533.

^f^ Adjacent to a gene of unknown function with transcript abundance higher in dichloromethane versus methanol.

^g^ Corresponding protein more abundant in cells grown with dichloromethane versus methanol (Muller et al., 2011).

**3. References**

Anders, S., McCarthy, D. J., Chen, Y., Okoniewski, M., Smyth, G. K., Huber, W., and Robinson M., D. (2013). Count-based differential expression analysis of RNA sequencing data using R and Bioconductor. *Nature protocols,*8,1765-1786. doi: 10.1038/nprot.2013.099.

Muller, E. E. L., Hourcade, E., Louhichi-Jelail, Y., Hammann, P., Vuilleumier, S., and Bringel, F. (2011). Functional genomics of dichloromethane utilization in *Methylobacterium extorquens* DM4. *Environ Microbiol* 13**,** 2518-2534. doi: 10.1111/j.1462-2920.2011.02524.x.

Roselli, S., Nadalig, T., Vuilleumier, S., and Bringel, F. (2013). The 380 kb pCMU01 plasmid encodes chloromethane utilization genes and redundant genes for vitamin B12- and tetrahydrofolate-dependent chloromethane metabolism in *Methylobacterium extorquens* CM4: a proteomic and bioinformatics study. *PloS ONE* 8**,** art. e56598. doi: 10.1371/journal.pone.0056598.

Vuilleumier, S., Chistoserdova, L., Lee, M.-C., Bringel, F., Lajus, A., Zhou, Y., et al. (2009). *Methylobacterium* genome sequences: a reference blueprint to investigate microbial metabolism of C1 compounds from natural and industrial sources. *PLoS ONE* 4**,** art. e5584. doi: 10.1371/journal.pone.0005584.
